# Sodium alginate-based dual-responsive nanoparticles co-loaded with baicalin and doxorubicin induce ferroptosis in hepatocellular carcinoma associated with the PI3K/AKT/FoxO3α axis regulation

**DOI:** 10.3389/fcell.2026.1905104

**Published:** 2026-09-03

**Authors:** Xining Li, Mingjie Bian, Jing Li

**Affiliations:** 1 School of Nursing and Medicine, Huzhou University, Huzhou, Zhejiang, China; 2 Neurobiology Laboratory, Wannan Medical College, Wuhu, Anhui, China

**Keywords:** baicalin, doxorubicin, ferroptosis, hepatocellular carcinoma, nanoparticles

## Abstract

**Background:**

Hepatocellular carcinoma (HCC) has high malignancy, poor prognosis, and frequent chemo-resistance. Doxorubicin (DOX) has strong anti-tumor activity but severe systemic toxicity; baicalin (BAI) acts against HCC yet has poor solubility and low bioavailability. Marine sodium alginate (SA) is a biocompatible carrier for responsive nanomedicines. We fabricated pH/GSH dual-responsive SA nanoparticles co-loaded with BAI and DOX (DOX/BAI NPs), tested synergistic anti-HCC activity, and verified the correlation between the PI3K/AKT/FOXO3α axis and DOX/BAI NPs-induced ferroptosis.

**Methods:**

Amphiphilic SA-SS-BAI was synthesized via cystamine disulfide bonds and self-assembled to encapsulate DOX. We characterized physicochemical traits, dual responsiveness, stability, and drug release. HepG2 cell uptake, endocytosis, and cytotoxicity were measured. Ferroptosis markers (ROS, Fe^2+^, GSH, MDA, SLC7A11, GPX4) were quantified. Western blot detected PI3K/AKT/FOXO3α activity, with SC79 rescue for mechanistic validation. Pharmacokinetics, anti-tumor efficacy and biosafety were evaluated in H22 tumor-bearing mice.

**Results:**

SA-SS-BAI-4 formed uniform ∼148 nm spherical DOX/BAI NPs with good loading and encapsulation. NPs stayed stable physiologically and disassembled rapidly under low pH plus high GSH. Caveolae-dependent uptake was greatly boosted in HepG2 cells, yielding stronger growth inhibition than free drugs or physical mixtures. NPs triggered robust ferroptosis via ROS/Fe^2+^ buildup, elevated MDA level, GSH loss, and reduced SLC7A11/GPX4. NPs treatment inhibited PI3K/AKT/FOXO3α phosphorylation; SC79 reversed ferroptosis, confirming the correlative regulatory relationship of this pathway. *In vivo*, NPs extended drug circulation, slowed H22 tumor growth, and markedly alleviated DOX-elicited hepatic and myocardial injury.

**Conclusion:**

This dual pH/GSH-responsive SA DOX/BAI nanoplatform exerts synergistic anti-HCC effects by inducing ferroptosis, which is associated with PI3K/AKT/FOXO3α axis suppression. The marine polysaccharide carrier overcomes BAI and DOX solubility/toxicity defects, creates a practical HCC combinatorial nanotherapy, and defines a potential correlation between the PI3K/AKT/FOXO3α regulatory axis and hepatic cancer ferroptosis.

## 1 Introduction

Hepatocellular carcinoma (HCC) is the most prevalent and lethal malignancy globally, characterized by high mortality rates and significant morbidity (Yang et al., 2023). Currently, chemotherapy remains the predominant treatment modality; however, commonly utilized anti-cancer agents exhibit poor water solubility and low bioavailability. This not only results in the destruction of tumor cells but also inflicts damage on adjacent normal cells (Zhang G et al., 2025). Prolonged administration of chemotherapeutic drugs can lead to multidrug resistance in cancer cells, thereby compromising clinical therapeutic efficacy (Wang Q et al., 2023). Consequently, the management of liver cancer continues to pose substantial challenges.

In recent years, natural bioactive compounds have gained increasing recognition as complementary anti-tumor agents. Combined use of natural bioactive molecules and chemotherapeutics has been proven to enhance anti-tumor efficacy via diverse molecular mechanisms (Li et al., 2025). Baicalin (BAI), a natural pharmacological compound derived from the root of Scutellaria baicalensis, is one of the primary bioactive components within flavonoids (Duan et al., 2019). It exhibits a broad spectrum of pharmacological activities, including anti-inflammatory, anti-allergic, antibacterial, antiviral, immunomodulatory, diuretic, and excretory effects (Shen et al., 2023). Furthermore, BAI has been reported to inhibit cancer cell proliferation, induce apoptosis and differentiation in cancer cells, and suppress cancer cell migration (Duan et al., 2019). However, BAI is characterized by poor solubility, low permeability, limited oral bioavailability, and rapid *in vivo* elimination, factors that restrict its clinical application (Zhang D et al., 2024). To address these challenges, numerous studies have developed an array of nanodelivery systems aimed at facilitating the transport of hydrophobic small molecule drugs.

Nano drug delivery systems have gained significant attention for their ability to enhance drug efficacy while minimizing toxic side effects (Chen et al., 2024). By assembling amphiphilic block copolymers into core-shell structures, hydrophobic drugs are encapsulated within the hydrophobic core, ensuring their stability. Furthermore, the hydrophilic shell enhances solubility and reduces protein adsorption on the carrier, thereby prolonging the circulation time of hydrophobic drugs in the bloodstream. This mechanism allows for effective evasion of recognition by the reticuloendothelial system and promotes selective accumulation of nanomedical drug delivery systems in tumor tissues, ultimately improving anti-tumor efficacy (Chen et al., 2024; Sun et al., 2023; Zhang J et al., 2024). Sodium alginate (SA) is characterized by a high density of hydroxyl groups and COO^−^ within its molecular structure, which imparts anionic polyelectrolyte properties in aqueous solutions, rendering sodium alginate pH-responsive (Sun et al., 2024). Drug delivery systems that incorporate pH-responsive chemical groups undergo structural transformations or break chemical bonds under acidic conditions, facilitating drug release. Cystamine (CYS) dihydrochloride, possessing a disulfide bond, enables the preparation of nanocarriers that are reductively responsive; these disulfide bonds can be cleaved to release drugs under the influence of glutathione (GSH) present in tumor cells (Zhang J et al., 2024).

Based on this, drug-carrying nanoparticles were synthesized in this study. BAI and SA were linked via disulfide bonds to create a pH/reduction dual-responsive polymer (SA-SS-BAI), with BAI serving as the hydrophobic end and SA as the hydrophilic end. The amphiphilic polymer self-assembled into nanoparticles in aqueous solution, encapsulating doxorubicin (DOX). Both BAI and DOX are contained within the nanoparticle core. Upon reaching the tumor site through the enhanced permeability and retention (EPR) effect, the SA molecular chain is extended, leading to the cleavage of disulfide bonds under acidic conditions and in response to GSH. This results in the release of internal anticancer agents, thereby facilitating a synergistic anticancer effect. In this experiment, HepG2 cells were selected as an *in vitro* model, while H22 tumor-bearing mice served as an animal model. Through both *in vivo* and *in vitro* experiments, we verified whether drug-loaded nanoparticles exhibit antitumor efficacy and analyzed their potential mechanisms of action.

## 2 Materials and methods

### 2.1 Materials

Dulbecco’s modified Eagle’s medium, fetal bovine serum, trypsin-EDTA solution, penicillin-streptomycin double antibiotics, and collagenase were purchased from Gibco Corporation (BRL, Gaithersburg, MD, United States). Hybond-C membrane and Western blot kits were obtained from Pierce Biotechnology (United States). 2′,7′-dichlorofluorescein diacetate (DCFH-DA) probe was supplied by Applygen Technologies Co., Ltd. The GSH assay kit was purchased from Nanjing Jiancheng Biotechnology Research Institute. Cell lysis buffer and BCA protein quantification kits were obtained from Shanghai Biyuntian Biotechnology Co., Ltd. All primary and secondary antibodies were acquired from Cell Signaling Technology. Enhanced chemiluminescence (ECL) reagent was purchased from Guangzhou Jabes Biotechnology Co., Ltd. DOX, BAI, calcein-acetoxymethyl ester (Calcein-AM), SC79 (PI3K activator), SA (MW ∼120 kDa), CYS, hexamethylenediamine, 4′,6-diamidino-2-phenylindole (DAPI), and 3-(4,5-dimethylthiazol-2-yl)-2,5-diphenyltetrazolium bromide (MTT) were obtained from Sigma-Aldrich (St. Louis, MO, United States). All chemicals and solvents were of analytical grade and used without further purification.

### 2.2 Preparation and characterization of nanoparticles

EDAC/N-NHS-mediated amide coupling chemistry was used for polymer synthesis. Synthesis of SS-SA polymer: Briefly, 100 mg SA powder was accurately weighed and dissolved in 20 mL ultrapure water under magnetic stirring (300 rpm) at room temperature for 2 h until complete dissolution. 120 mg EDAC was added into the SA aqueous solution. After 45 min activation under magnetic stirring, 72 mg NHS was supplemented under nitrogen-protected dark conditions, and the mixture was continuously stirred overnight (12 h) to fully activate the carboxyl groups of SA. Subsequently, 80 mg CYS was dissolved in 10 mL ultrapure water, and this CYS solution was mixed with the activated SA solution. The reaction proceeded at room temperature with magnetic stirring (300 rpm) for 48 h to obtain crude SS-SA product. The crude reaction mixture was transferred into a dialysis bag (MWCO = 8,000 Da) and dialyzed against ultrapure water for 48 h; the external water was renewed every 6 h. After lyophilization under vacuum, purified SS-SA polymer powder was stored at 4 °C in dark for subsequent use.

Synthesis of SA-SS-BAI conjugate: 50 mg lyophilized SS-SA polymer was dissolved in 15 mL formamide under stirring to prepare SS-SA stock solution. According to feeding ratios summarized in Table 1; for SA-SS-BAI-4, 25 mg BAI powder was weighed, mixed with 30 mg EDAC and 18 mg NHS, and dissolved in 10 mL N,N-dimethylformamide (DMF). This BAI mixture was magnetically stirred for 2 h at room temperature in dark for carboxyl activation. The activated BAI-DMF solution was drop-wise added into SS-SA formamide solution, and the whole system was stirred (300 rpm) at room temperature in dark for 48 h to generate crude SA-SS-BAI conjugate. The crude product was dialyzed against ultrapure water for 3 days with external water renewed every 8 h, lyophilized under vacuum, and stored at 4 °C away from light. Determination of critical micelle concentration (CMC): CMC of SA-SS-BAI was measured via pyrene fluorescence probe assay. Pyrene ethanol stock solution (12 μg/mL) was prepared. A certain volume of pyrene stock solution was added into series of glass tubes, and ethanol was completely evaporated under vacuum. Serial-diluted SA-SS-BAI aqueous solutions were added to each tube to obtain final polymer concentrations ranging from 0.01 mg/mL to 0.2 mg/mL. Each mixture was sonicated for 30 min in ice-water bath and incubated in darkness at room temperature for 8 h. Fluorescence spectrometer parameters: excitation wavelength = 334 nm, emission spectra were recorded from 300 nm to 700 nm. CMC value was calculated from the abrupt change of pyrene fluorescence intensity ratio.

**TABLE 1:** Substitution degree, BAI grafting ratio and CMC of SS-SA-BAI polymers prepared at different SS-SA/BAI feeding ratios.

Sample	SS-SA/BAI feeding ratio	BAI grafting ratio (%)	CMC/(mg/mL)
SA-SS-BAI-1	10:1	4.12 ± 0.31	No nanoparticles were generated
SA-SS-BAI-2	10:2	7.35 ± 0.44	No nanoparticles were generated
SA-SS-BAI-3	10:4	12.68 ± 0.57	0.063
SA-SS-BAI-4	10:5	14.23 ± 0.62	0.051

Fabrication of DOX/SA-SS-BAI-4 nanoparticles: 10 mg lyophilized SA-SS-BAI-4 powder was dispersed in 20 mL ultrapure water under magnetic stirring (400 rpm) as aqueous phase. 3 mg DOXHCl was dissolved in 2 mL methanol; triethylamine (2-fold molar excess relative to DOX, 1.2 μL) was added to deprotonate DOX. The mixture was stirred in dark for 2 h at room temperature. Methanol solvent was completely removed by vacuum rotary evaporation at 35 °C. The residual DOX solid was redissolved in 3 mL dichloromethane to form organic phase. Organic phase was drop-wisely injected into the above aqueous phase at a dropping rate of 1 mL/min under vigorous stirring (600 rpm). The mixed emulsion system was continuously stirred in darkness for 24 h. The crude nanoparticle suspension was dialyzed against ultrapure water for 2 days (MWCO = 8,000 Da, water renewed every 12 h), sonicated in ice-water bath for 20 min, and filtered through a 0.45 μm mixed cellulose ester membrane to harvest final DOX/BAI NPs suspension.

Quantification of DOX encapsulation efficiency (EE) and drug-loading content (DLC): 1 mL freshly prepared nanoparticle suspension was mixed with 4 mL methanol and disrupted by probe sonication (300 W, 10 min, ice-bath). DOX concentration was quantified by high-performance liquid chromatography (HPLC). EE and DLC were calculated according to following formulas:

$$\text{EE }\left(\%\right)=\left(\text{mass of encapsulated DOX}/\text{total feeding DOX mass}\right)\times 100\%$$

$$\text{DLC }\left(\%\right)=\left(\text{mass of encapsulated DOX}/\text{mass of lyophilized nanoparticles}\right)\times 100\%.$$

Nanoparticle morphology was observed by transmission electron microscopy (Tecnai G2-20, 200 kV). Particle-size distribution and zeta-potential were measured by dynamic light scattering (DLS) instrument (Wyatt Technology, United States) equipped with vertically polarized He-Ne laser at 25 °C.

Determination of BAI grafting ratio: Lyophilized SA-SS-BAI polymer powders were repeatedly washed with methanol three times to thoroughly remove physically adsorbed free BAI and vacuum-dried at room temperature. 5 mg pre-treated polymer was accurately weighed and fully dissolved in 5 mL pH 7.4 PBS buffer. Blank SS-SA polymer solution at equal mass concentration was set as reference control. UV-Vis absorbance was detected at 278 nm (maximum absorption wavelength of BAI). The mass of covalently conjugated BAI was calculated referring to pre-established BAI standard curve.

$$\text{Grafting ratio }\left(\%\right)=\left(\text{mass of covalently conjugated BAI}/\text{total mass of SA}-\text{SS}-\text{BAI polymer}\right)\times 100\%.$$

Three independent replicate samples were tested, and data were presented as mean ± SD. BAI grafting ratios for each polymer group were summarized in Table 1.

### 2.3 Stability evaluation

Lyophilized DOX/SA-SS-BAI-4 nanoparticle (DOX/BAI NPs) powder was weighed and homogeneously dispersed in ultrapure water by sonication to obtain initial nanoparticle suspension. The suspension was stored at 4 °C in dark for 30 days. Aliquots were collected at predetermined time-points: day 0, 1, 3, 7, 10, 15, 20, 25, and 30. Particle-size changes were measured by DLS at 25 °C. Each time-point measurement was performed in triplicate to assess colloidal stability.

### 2.4 Investigation of stimulus response

Particle-size distribution and polydispersity index (PDI) of nanoparticles under pH and GSH stimuli were assessed by DLS. Briefly, 1 mL DOX/BAI NPs suspension was mixed with 9 mL of four different buffer systems: pH 5.0 PBS containing 10 μM GSH; pH 5.0 PBS containing 10 mM GSH; pH 7.4 PBS containing 10 μM GSH; pH 7.4 PBS containing 10 mM GSH. A control sample was prepared by mixing 1 mL nanoparticle suspension with 9 mL ultrapure water. All samples were incubated at 37 °C for 4 h before DLS measurement at 25 °C; particle-size distribution profiles and PDI values were recorded for each condition.

### 2.5 *In vitro* evaluation of stimulus-responsive drug release

*In vitro* dual-drug release behavior was investigated using dynamic dialysis method. The release media included pH 5.0 PBS/10 μM GSH, pH 7.4 PBS/10 μM GSH, pH 5.0 PBS/10 mM GSH, and pH 7.4 PBS/10 mM GSH. 2 mL DOX/BAI NPs suspension (final content: 0.5 μg DOX equivalent and molar-matched corresponding BAI) was sealed inside dialysis bag (MWCO = 3,500 Da). The dialysis bag was immersed into 40 mL pre-warmed corresponding release buffer. The whole system was incubated in constant-temperature oscillator set at 37 °C with shaking speed 150 rpm. At each designated sampling time-point (0, 2, 4, 8, 12, 16, 24, 30, 36 h), 1 mL release medium was withdrawn, and immediately replaced with 1 mL pre-warmed fresh corresponding buffer to maintain constant volume. The concentrations of DOX and BAI in collected release medium were quantified by HPLC according to pre-calibrated standard curves. All release experiments were performed with n = 3 independent biological replicates. Cumulative release percentages of DOX and BAI were calculated for each time-point.

### 2.6 Cell uptake

HepG2 cells were seeded in 6-well plates at a density of 1 × 10^6^ cells per well with sterile glass coverslips placed inside each well. Cells were cultured in complete DMEM medium for 24 h for attachment. Afterwards, culture medium was replaced with fresh medium containing different formulations: free DOX, free DOX + free BAI physical mixture, DOX/BAI NPs. The final working concentration of DOX was 10 μg/mL for all DOX-containing groups; BAI was added at molar-matched concentration corresponding to 10 μg/mL DOX. After 2 h incubation at 37 °C, 5% CO_2_, cells were rinsed three times with pre-cold PBS, fixed with 4% paraformaldehyde aqueous solution for 10 min at room temperature. Nuclei were stained with DAPI working solution. Coverslips were mounted onto glass slides, and cellular fluorescence signals were visualized by confocal laser scanning microscope (CLSM, Olympus, Tokyo, Japan).

### 2.7 Flow cytometry

Endocytosis mechanism of DOX/BAI NPs in HepG2 cells was analyzed by flow cytometry. HepG2 cells were seeded in 6-well plates at density of 5 × 10^4^ cells per well and incubated in complete DMEM medium for 24 h. Six experimental groups were set with three replicate wells per group. Groups 1–4 were pre-incubated for 1 h at 37 °C with endocytosis inhibitors at final working concentrations: chlorpromazine 10 μg/mL, quercetin 40 μg/mL, indomethacin 100 μg/mL, β-cyclodextrin 2 mg/mL, respectively. Subsequently, DOX/BAI NPs were added into each well together with corresponding inhibitors and incubated for another 1 h at 37 °C. Group 5: cells were pre-incubated at 4 °C for 1 h without inhibitors, then treated with DOX/BAI NPs and incubated at 4 °C for another 1 h. Group 6 served as normal temperature control: cells received DOX/BAI NPs treatment and incubated at 37 °C. For all groups, final DOX working concentration was 10 μg/mL, BAI concentration was molar-matched to DOX. After incubation, cells were washed twice with PBS, digested by trypsin-EDTA, collected by centrifugation at 5,000 rpm for 5 min. Cell pellets were washed twice with ice-cold PBS, and cell suspensions were analyzed by flow cytometer (Coulter FC500, Beckman, Piscataway, NJ, United States). A total of 10,000 gated cellular events were acquired for each sample; data were analyzed using ModFit LT 3.0 software.

### 2.8 Cell proliferation assay

HepG2 cells in logarithmic growth phase were seeded into 96-well plates at density of 1 × 10^4^ cells per well; each well contained 100 μL complete DMEM medium. Cells were cultured at 37 °C, 5% CO_2_ for 24 h until full adhesion. Original medium was discarded, and cells were treated with serial diluted drug formulations for 48 h. Treatment groups: Group I: free BAI; Group II: free DOX; Group III: physical mixture of free BAI + free DOX; Group IV: DOX/BAI NPs. Final DOX equivalent working concentrations were set as 0.25 μg/mL, 0.5 μg/mL, 1 μg/mL, 5 μg/mL, 10 μg/mL. For each concentration point, BAI was supplemented at strictly molar-matched concentration corresponding to each DOX concentration. Six replicate wells were set for every concentration. After 48 h drug incubation, culture medium was removed, each well was washed twice with PBS. 20 μL MTT working solution (5 mg/mL in PBS) was added to each well, and plates were incubated at 37 °C for 4 h. MTT-containing medium was discarded, 150 μL dimethyl sulfoxide was added per well to fully dissolve formazan crystals. Absorbance value at 490 nm was measured by microplate reader. Three technical readings were collected for each well, and average absorbance was used to calculate relative cell viability.

### 2.9 ROS detection

Intracellular ROS levels in HepG2 cells were determined using DCFH-DA fluorescent probe. HepG2 cells were seeded into 24-well plates at density of 1 × 10^4^ cells per well with sterile glass coverslips inside each well. After cell adhesion, cells were exposed to different drug formulations for 48 h (DOX final working concentration = 10 μg/mL, BAI molar-matched). After treatment, cells were washed twice with PBS and incubated with DCFH-DA working solution (final concentration = 10 μM) at 37 °C for 30 min in dark. Nuclei were counter-stained with DAPI. Green fluorescence signals were observed under fluorescence microscope (Olympus, Tokyo, Japan).

### 2.10 Fe^2+^ quantification in HepG2 cells

Intracellular Fe^2+^ was detected via Calcein-AM fluorescence quenching assay. HepG2 cells were seeded in 6-well plates at density of 1 × 10^6^ cells per well with glass coverslips. After attachment, cells were treated with different drug formulations for 48 h (DOX final working concentration = 10 μg/mL, BAI molar-matched). Culture medium was discarded, wells were rinsed once with PBS. 500 μL Calcein-AM working solution (final concentration = 20 μM) was added into each well, incubated at 37 °C for 30 min in dark. Nuclei were stained with DAPI, and excess probe was washed away twice with PBS. Fluorescence quenching signals were observed under fluorescence microscope (Olympus, Tokyo, Japan).

### 2.11 GSH detection

Intracellular GSH content in HepG2 cells was measured using commercial GSH biochemical assay kit strictly following manufacturer’s protocols. Briefly, after 48 h drug treatment, cells were collected and lysed. Cell lysates and series of GSH standard samples were processed in parallel. Absorbance was read at 450 nm using microplate reader. Intracellular GSH concentrations were calculated according to pre-established standard calibration curve.

### 2.12 Immunofluorescence analysis

HepG2 cells in logarithmic growth phase were seeded in 6-well plates at density of 1 × 10^6^ cells per well with glass coverslips. After adhesion, cells were treated with drugs for 48 h (DOX final working concentration = 10 μg/mL, BAI molar-matched). Cells were washed with PBS, fixed in 4% paraformaldehyde for 20 min at room temperature, and blocked with 1% bovine serum albumin-PBS solution at room temperature for 30 min. Primary antibody incubation was performed overnight at 4 °C. After TBST washing, secondary antibody was incubated at room temperature for 1.5 h in dark. Nuclei were counter-stained with DAPI. Intracellular target-protein fluorescence intensity was observed under fluorescence microscope (Olympus IX71, Japan).

### 2.13 Determination of intracellular malondialdehyde (MDA) level

Intracellular MDA content was determined using commercial MDA assay kit strictly following the manufacturer’s instructions. Briefly, HepG2 cells received different drug treatments for 48 h (DOX final working concentration = 10 μg/mL, BAI molar-matched). After treatment, cells were harvested and lysed in cell lysis buffer on ice. Cell lysate supernatant was collected after centrifugation, and MDA content was detected according to kit protocols. The absorbance was measured at 532 nm using a microplate reader. Intracellular MDA concentration was calculated based on the standard calibration curve. Three independent biological replicates were performed, and data were presented as mean ± SD.

### 2.14 Western blotting assay

HepG2 cells were seeded in cell culture dishes at density of 1 × 10^6^ cells per dish. After cell adhesion, cells were treated with different drug formulations for 48 h (DOX final working concentration = 10 μg/mL, BAI molar-matched). Cells were washed twice with pre-cold PBS, scraped and collected into centrifuge tubes, centrifuged at 12,000 rpm, 4 °C for 10 min. Cell pellets were resuspended in 100 μL RIPA lysis buffer supplemented with protease-phosphatase inhibitor cocktail, lysed on ice for 30 min, and further centrifuged at 12,000 rpm, 4 °C for 10 min. Supernatant was collected as total protein sample. Total protein concentration was quantified by BCA assay kit. 20 μL 5×SDS loading buffer was mixed with protein sample, and total volume was adjusted to 100 μL using double-distilled water. Samples were boiled for 10 min and cooled to room temperature. Equal amount of total protein (∼50 μg) per lane was loaded for SDS-PAGE electrophoresis (constant voltage 100 V, 80 mA). After electrophoresis, proteins were electro-transferred onto 0.45 μm PVDF membranes under conditions: 25 V, 1 A. Membranes were blocked with 5% non-fat dry milk-TBST solution for 1 h at room temperature. Primary antibodies were incubated overnight at 4 °C. After three TBST washes, secondary antibodies were incubated for 1 h at room temperature. After another three TBST washes, protein bands were visualized with ECL reagent on gel imaging analysis system. Band grey-scale intensities were quantified by ImageJ 1.8.0 software. β-actin was used as internal reference for normalization of relative target-protein expression.

### 2.15 *In vivo* pharmacokinetics

After H22 tumor-bearing mouse model establishment, mice were randomly divided into four groups. Free DOX, free BAI, DOX + BAI physical mixture, or DOX/BAI NPs were administrated via tail-vein injection at equivalent DOX dose. Blood samples were collected at predetermined time-points (0.08, 0.16, 0.5, 1, 2, 3, 6, 9, 12, 24, 48 and 72 h) using heparin-coated centrifuge tubes. Plasma fractions were obtained by centrifugation at 4,000 rpm for 10 min at 4 °C. Plasma proteins were precipitated with methanol, supernatant was collected and analyzed by UPLC-MS/MS.

### 2.16 *In vivo* experiments

All animal experiments complied with the National Institutes of Health Guidelines for the Care and Use of Laboratory Animals and were approved by the Animal Experiment Ethics Committee of Wannan Medical College. H22 hepatoma cells were cultured in RPMI-1640 medium supplemented with 10% fetal bovine serum and 1% penicillin-streptomycin. Logarithmic-phase H22 cells were collected, washed with sterile PBS, and resuspended in sterile normal saline at cell density of 1 × 10^7^ cells/mL. Each mouse received 0.2 mL cell suspension by intraperitoneal injection. When obvious ascites formed in peritoneal cavity, ascitic H22 tumor cells were aseptically harvested, washed, resuspended and adjusted to cell density of 2 × 10^7^ cells/mL. 0.1 mL cell suspension was subcutaneously inoculated into the right forelimb axilla of each mouse. Tumor length (a) and width (b) were measured by vernier caliper every 2 days; tumor volume (TV) was calculated as TV = (1/2) × a × b^2^. When subcutaneous tumor volume reached 100–150 mm^3^ (7 days post-inoculation), tumor-bearing model was considered successfully established. Mice received tail-vein administration every 3 days. Mental status, body weight and tumor volume were recorded throughout treatment period. After 11 days of treatment, mice were euthanized by cervical dislocation. Tumor inhibition rate (TIR) was calculated using terminal tumor volume at day 11:

TIR (%) = (1−mean tumor volume of treatment group/mean tumor volume of control group) × 100%.

After euthanasia, liver and myocardial tissues were dissected, fixed in 4% paraformaldehyde for 24 h, paraffin-embedded, cut into 5 μm continuous sections, and stained with hematoxylin-eosin (HE). Histopathological slices were imaged under optical microscope at ×200 magnification (scale bar = 50 μm). Histopathological alterations including inflammatory infiltration, cellular edema, vacuolar degeneration, necrosis, myocardial-fiber disorganization and interstitial edema were blindly evaluated by two independent pathologists.

Orbital whole-blood samples were collected after euthanasia, centrifuged at 4,000 rpm for 10 min at 4 °C to obtain serum supernatant. Serum myocardial injury markers CK-MB, cTn-I and hepatic injury indicators ALT, AST were measured by commercial ELISA kits strictly following manufacturer’s instructions. Absorbance values were read with microplate reader, and biomarker concentrations were calculated from corresponding standard curves. Each serum sample was detected in duplicate; n = 5 biological replicates per group.

### 2.17 Statistical analysis

All experimental data are expressed as mean ± standard deviation (SD). *In vitro* cell and molecular experiments were performed with n = 3 independent replicates. For *in vivo* animal assays, n = 5 biological replicates per group, except for pharmacokinetic samples. Multi-group comparisons were performed using one-way ANOVA followed by Tukey’s *post hoc* multiple comparison test. P < 0.05 was considered statistically significant. Tumor inhibition rate was calculated based on terminal tumor volume at day 11 of treatment.

## 3 Results

### 3.1 Characterization of nanoparticles

As presented in Table 1, At feeding ratios of 10:1 and 10:2, the obtained SA-SS-BAI-1 and SA-SS-BAI-2 failed to form nanoparticles. This result suggests insufficient BAI grafting led to inadequate hydrophobicity, which impeded nanoparticle assembly. In contrast, nanoparticles of SA-SS-BAI-3 and SA-SS-BAI-4 were successfully generated, with CMC values showed a decreasing trend. This indicates that an increased degree of BAI grafting onto the amphiphilic polymer enhances its hydrophobic effect and improves its ability to form structures. Based on these comparisons, SA-SS-BAI-4 was selected for subsequent experiments.

**FIGURE 1:**
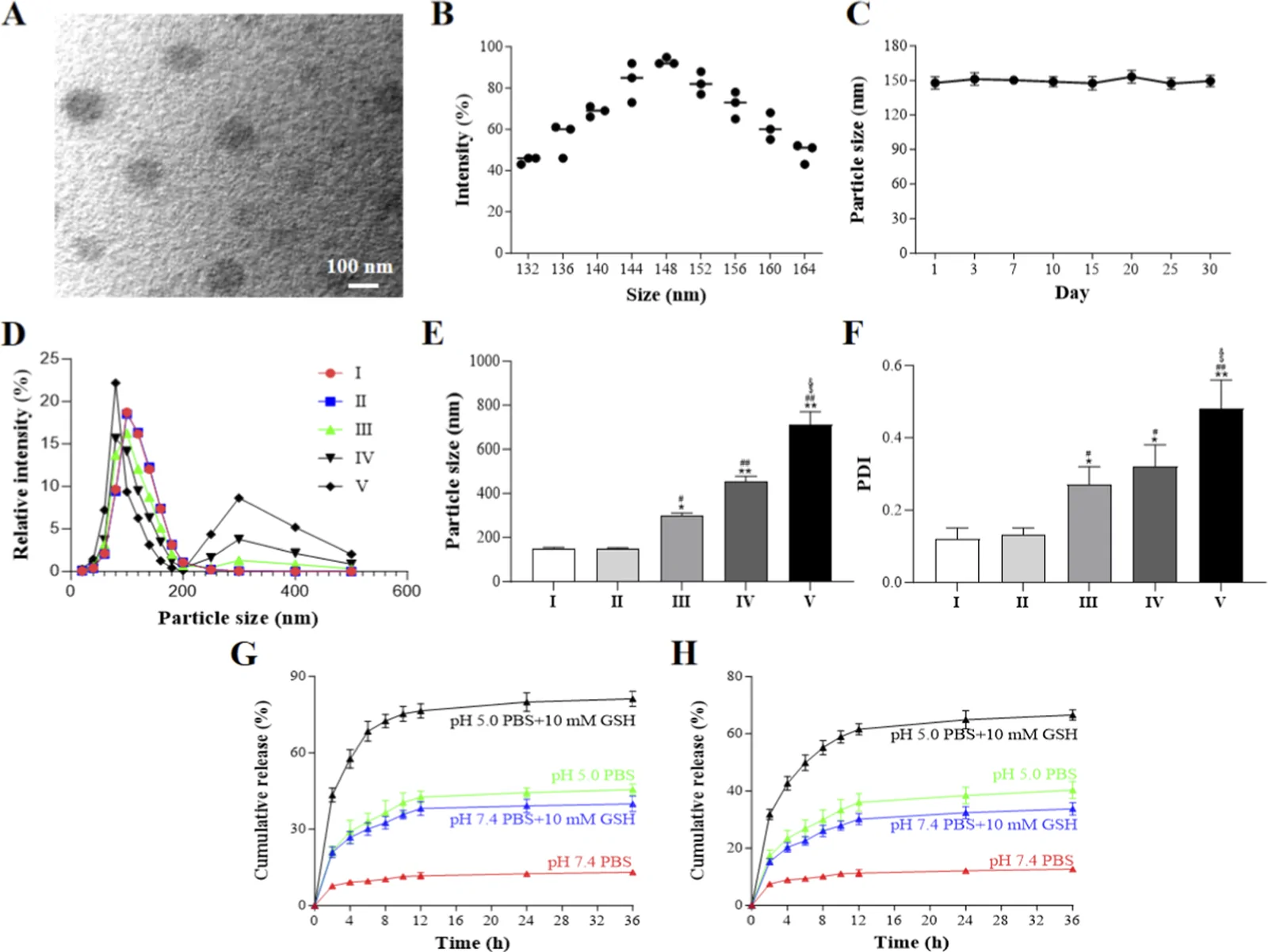
Characteristics of DOX/BAI NPs. **(A)** Representative TEM micrograph of DOX/BAI NPs, scale bar = 100 nm. **(B)** Hydrodynamic particle-size distribution of DOX/BAI NPs determined by DLS. **(C)** Long-term colloidal stability of DOX/BAI NPs; particle size was continuously monitored over 30 days. **(D–F)** Changes in particle-size distribution. **(D)**, average particle size. **(E)**, and PDI **(F)** of DOX/BAI NPs under different stimulus conditions. I: Ultrapure water; II: pH 7.4 PBS; III: pH 7.4 PBS containing 10 mM GSH; IV: pH 5.0 PBS; V: pH 5.0 PBS containing 10 mM GSH. *p < 0.05, **p < 0.01 vs. I group; #p < 0.05, ##p < 0.01 vs. II group; $ p < 0.05 vs. III group; and p < 0.05 vs. IV group. **(G,H)** *In vitro* cumulative drug-release profiles of DOX/BAI NPs under identical pH and GSH stimulus conditions. **(G)** Cumulative release of DOX. **(H)** Cumulative release of BAI. Data are shown as mean ± SD, n = 3 for all *in vitro* experiments.

After measurement and calculation, the drug loading capacity of DOX/BAI NPs was found to be (17.26 ± 0.12) %, while the encapsulation efficiency was determined to be (64.28 ± 0.29) %. As illustrated in Figure 1A, DOX/BAI NPs exhibit a spherical morphology with a uniform distribution and small particle size. Figure 1B presents data from DLS, indicating that the particle size of DOX/BAI NPs is 148 nm, with a polydispersity index of (0.12 ± 0.03), and a zeta potential of -(23.16 ± 0.23) mV. Based on these findings, we conclude that the drug-carrying nanoparticles developed in this study possess ideal characteristics such as appropriate size, high dispersion stability, and favorable physical and chemical parameters, thereby demonstrating significant potential for practical applications. As shown in Figure 1C, results from stability tests indicate that the particle size of DOX/BAI NPs remains relatively unchanged over time, suggesting that these nanoparticles can maintain a stable state at 4 °C.

The DOX/BAI NPs prepared in this study possess carboxyl groups and cleavable disulfide bonds, enabling the nanoparticles to undergo cleavage under acidic and reducing conditions. As shown in Figure 1D, DLS particle-size distribution curves directly visualized the structural variation of DOX/BAI NPs under different stimulus treatments. Under ultrapure water (Group I) and pH 7.4 PBS (Group II), nanoparticles maintained a narrow unimodal size distribution. Obvious broadening and right-shift of particle-size distribution were observed upon single-stimulus treatment: either high GSH (pH 7.4, 10 mM GSH, Group III) or acidic condition (pH 5.0 PBS, Group IV). A marked shift toward larger particle size and multimodal distribution occurred under combined pH 5.0 + 10 mM GSH (Group V), indicating severe nanoparticle disassembly and aggregation. Consistently, Figure 1F showed that PDI values remained low in control groups, while PDI increased significantly upon single-stimulus exposure and reached the highest level under combined low-pH and high-GSH conditions. These results collectively demonstrate that the dual stimuli of acidic pH and high intracellular GSH synergistically trigger the disassembly of DOX/BAI NPs.

The *in vitro* drug release profiles of DOX/BAI NPs are shown in Figures 1G,H. The DOX release curves are presented in Figure 1G, and BAI release curves are shown in Figure 1H. DOX exhibited the highest release rate at pH 5.0 with 10 mM GSH, achieving a cumulative release of 81.2% within 36 h. Under the conditions of pH 5.0 with 10 μM GSH and pH 7.4 with 10 mM GSH, cumulative DOX releases after 36 h were 45.63% and 39.97%, respectively. Under pH 7.4 with 10 μM GSH, minimal DOX release (13.17%) was detected. Consistent with DOX, BAI displayed typical pH/GSH dual-responsive release (Figure 1H), with maximum cumulative release under combined pH 5.0 and 10 mM GSH conditions, while negligible release was observed under physiological-mimicking pH 7.4/10 μM GSH environment. These findings further corroborate that optimal payload release from DOX/BAI NPs occurs under combined acidic pH and elevated GSH conditions, consistent with the DLS stimulus-response observations in Figures 1D–F. Consistent with the DLS observations of particle size enlargement and multimodal distribution under combined acidic and reductive conditions, the dramatically accelerated drug release further supports that low-pH and high-GSH microenvironment triggers the disassembly of the polymeric nanoparticle architecture. The combined evidence from hydrodynamic size shift, elevated PDI, and stimulus-dependent drug release collectively indicates that pH-triggered alginate chain extension together with disulfide bond cleavage leads to nanoparticle structural disintegration, which ultimately drives payload liberation.

### 3.2 Cell uptake and mechanism of DOX/BAI NPs

**FIGURE 2:**
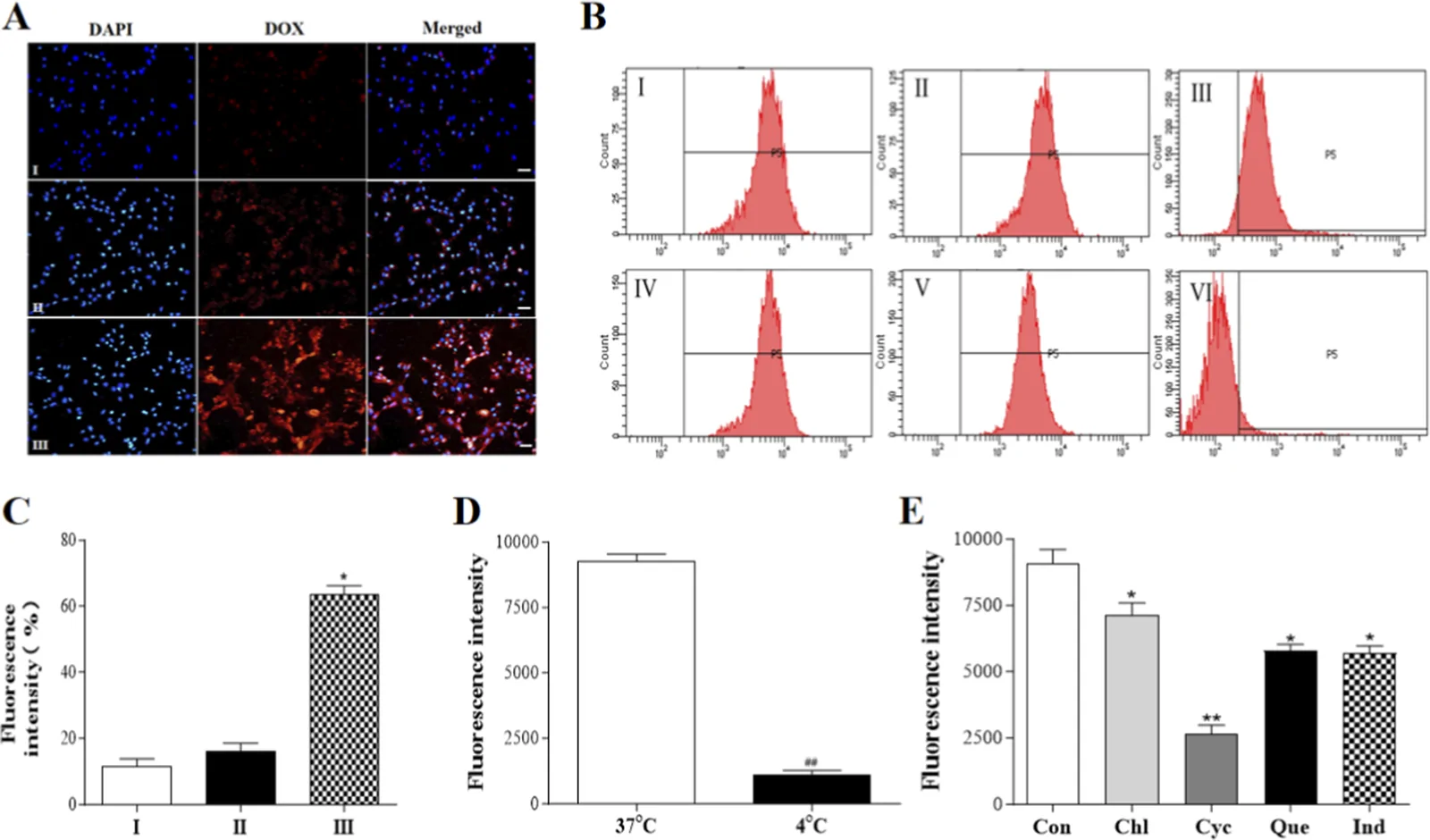
Cellular uptake and endocytosis mechanism of DOX/BAI NPs in HepG2 cells. **(A)** Fluorescence microscopy images showing the cellular uptake of DOX/BAI NPs in HepG2 cells. Blue fluorescence (DAPI) indicates cell nuclei, and red fluorescence (DOX) indicates the intracellular distribution of nanoparticles. Scale bar = 20  μm *p < 0.05 vs. DOX group. **(B)** Flow cytometry analysis of DOX fluorescence intensity in HepG2 cells treated with DOX/BAI NPs under different conditions: I: 37 °C, control (no inhibitor pretreatment); II: 37 °C, pretreatment with chlorpromazine; III: 37 °C, pretreatment with β-cyclodextrin; IV: 37 °C, pretreatment with quercetin; V: 37 °C, pretreatment with indomethacin; VI: 4 °C (low temperature inhibition, no inhibitor pretreatment). The P5 label indicates the instrument-defined gate for positive fluorescence events. **(C)** Quantification of mean fluorescence intensity from panel A, showing the relative cellular uptake efficiency across groups. *p < 0.05 vs. I group. **(D)** Comparison of average DOX fluorescence intensity in cells incubated at 37 °C versus 4 °C, demonstrating the temperature dependence of endocytosis. **p < 0.01 vs. 37 °C control group. **(E)** Effects of different endocytosis inhibitors on DOX/BAI NPs uptake in HepG2 cells. Con: control; Chl: chlorpromazine; Cyc: β-cyclodextrin; Que: quercetin; Ind: indomethacin. Data are presented as mean ± SD, n = 3 for all *in vitro* experiments. *p < 0.05, **p < 0.01 vs. control group.

In order to observe the cell uptake, HepG2 cells were incubated with free DOX, DOX + BAI physical mixture, and DOX/BAI NPs for 2 h, and cellular uptake was visualized using CLSM. Figure 2A shows that the cells in the DOX group absorbed very little red fluorescence, and the red fluorescence in the DOX + BAI physical mixing group was higher than that in the DOX group. When the cells were treated with DOX/BAI NPs, the uptake of DOX was greatly enhanced, and the red fluorescence was significantly stronger than that in the DOX group or the DOX + BAI physical mixing group. As shown in Figure 2C, the bar graph data once again confirmed that the uptake of DOX/BAI NPs by HepG2 cells was significantly stronger than that in the small molecule drug group or the physical mixing group.

HepG2 cells without pretreatment with endocytosis inhibitors were incubated with DOX/BAI NPs at two different temperatures of 37 °C and 4 °C to observe the difference in DOX/BAI NPs uptake. Typical flow cytometry patterns for these two conditions are shown in Figure 2B. It was observed that when the culture temperature was set at 37 °C and 4 °C, there was a significant difference in the endocytosis of DOX/BAI NPs (these two conditions were I and VI in Figure 2B, respectively). More samples cultured at 37 °C and 4 °C were measured, and the quantitative analysis of mean fluorescence intensity is presented in Figure 2D. The bar graph in Figure 2D showed that the endocytosis of DOX/BAI NPs at 37 °C was about 8.2 times higher than that measured at 4 °C, confirming that the endocytosis pathway of DOX/BAI NPs in HepG2 cells is highly energy dependent. To ensure efficient cellular uptake of DOX/BAI NPs, all subsequent cell experiments were performed at 37 °C. HepG2 cells pretreated with four types of endocytosis inhibitors (chlorpromazine, β-cyclodextrin, quercetin, and indomethacin) were incubated with DOX/BAI NPs at 37 °C to identify possible uptake mechanisms, and the quantitative mean fluorescence intensity data are presented in Figure 2E (these four cases were II, III, IV, and V, respectively). In order to quantitatively compare the effects of endocytosis inhibitors on DOX/BAI NPs uptake, more samples corresponding to each inhibitor were measured, and the mean fluorescence intensity was shown in Figure 2E. These results indicated that cellular uptake of DOX/BAI NPs is energy-dependent and mainly relies on caveolae-mediated endocytosis, while clathrin-mediated pathways play a minor role.

### 3.3 Proliferation inhibitory effects on hepatoma and normal hepatic cells

**FIGURE 3:**
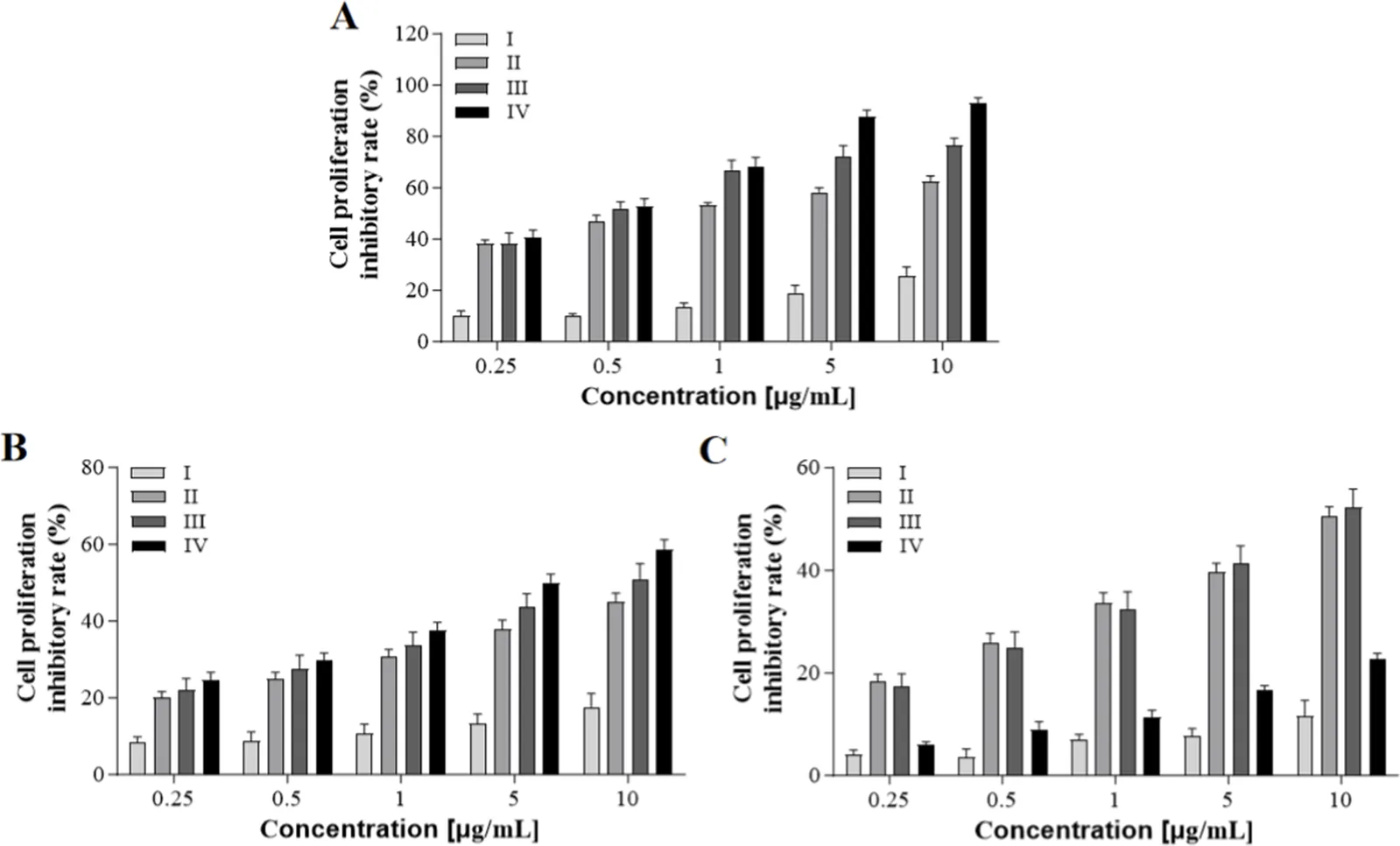
Impact of DOX/BAI NPs on cell proliferation. **(A)** HepG2 cells. **(B)** Huh7 cells. **(C)** LO2 normal hepatocytes. I: BAI; II: DOX; III: BAI + DOX; IV: DOX/BAI NPs. Data are presented as mean ± SD, n = 3. Full pairwise comparison p-values are provided in Supplementary Tables S1-S3.

The anti-proliferative activities of free BAI, free DOX, BAI + DOX physical mixture, and DOX/BAI NPs were evaluated in two hepatocellular carcinoma cell lines (HepG2, Huh7) and normal human hepatocyte LO2 cells. For HepG2 cells (Figure 3A), all drug groups showed dose-dependent proliferation inhibition. At DOX equivalent concentrations higher than 1 μg/mL, both the BAI + DOX physical-mixture group and DOX/BAI NPs group exhibited significantly stronger inhibitory effects compared with free BAI and free DOX. When the DOX equivalent exceeded 5 μg/mL, DOX/BAI NPs achieved higher inhibition rate than the BAI + DOX physical mixture, demonstrating the superiority of the nanocarrier delivery system. Huh7 cells displayed similar dose-response trends (Figure 3B). The inhibitory potency followed the rank: DOX/BAI NPs > BAI + DOX physical mixture > free DOX >> free BAI, confirming that DOX/BAI NPs could efficiently suppress proliferation of another hepatoma cell line. In contrast, distinct results were observed in LO2 normal hepatocytes (Figure 3C). Free DOX and BAI + DOX mixture caused obvious cytotoxic injury to normal liver cells. Nevertheless, DOX/BAI NPs exerted only limited growth-inhibitory effect against LO2 cells even at higher concentrations. These findings suggest DOX/BAI NPs possess favorable tumor-selective cytotoxicity and reduced toxicity toward normal hepatocytes. Based on the above results, 5 μg/mL (DOX equivalent) was selected as the working concentration for subsequent *in vitro* experiments.

### 3.4 Investigation of ferroptosis in HepG2 cells

**FIGURE 4:**
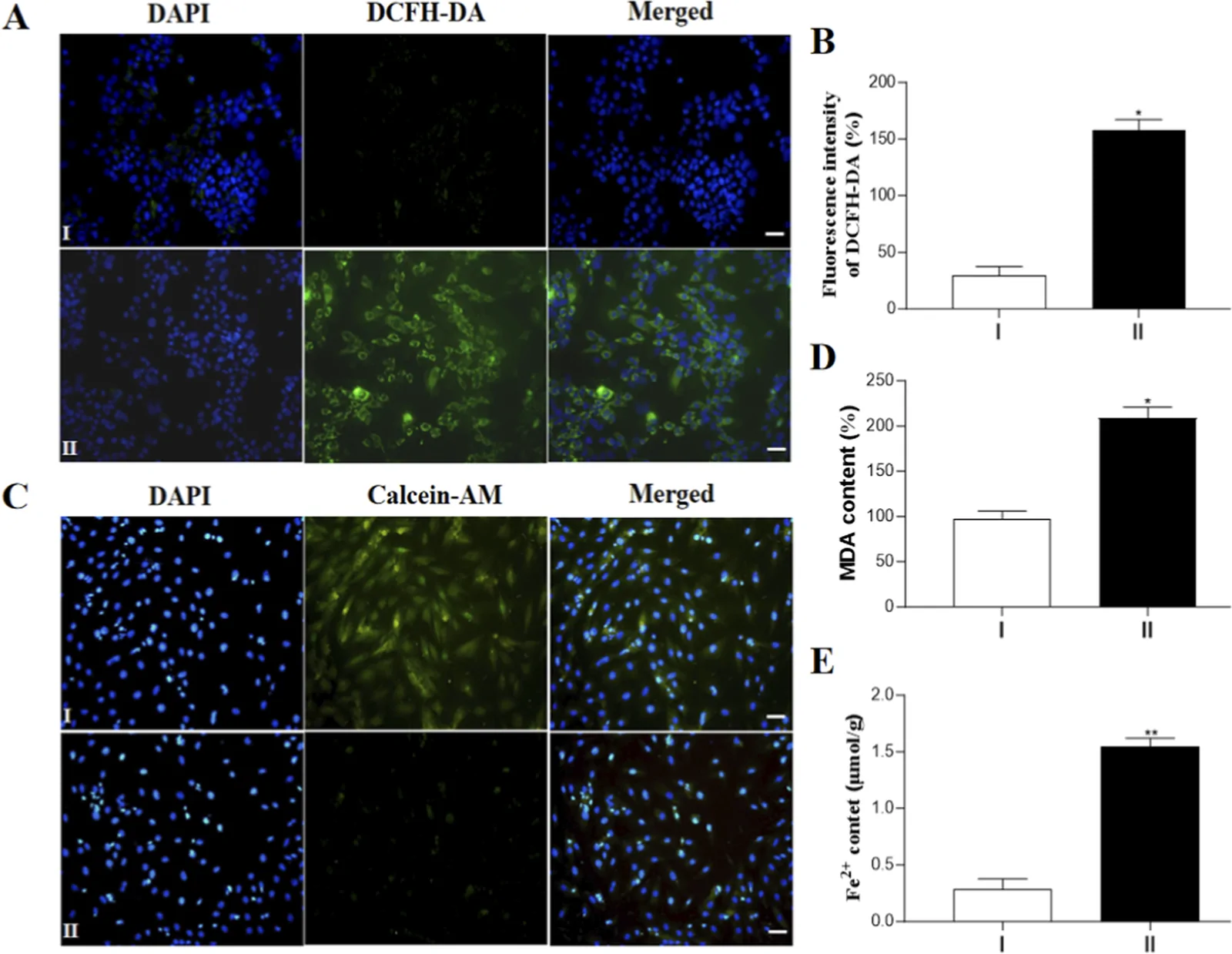
Effects of DOX/BAI NPs on intracellular ROS and Fe^2+^ levels in HepG2 cells. **(A)** CLSM images of DAPI- and DCFH-DA-stained cells for ROS detection. Scale bar = 20 μm. **(B)** Quantitative analysis of DCFH-DA fluorescence intensity. **(C)** CLSM images of DAPI- and Calcein-AM-stained cells for Fe^2+^ detection (Calcein-AM quenching assay). Scale bar = 20 μm. **(D)** Quantification of intracellular MDA content. **(E)** Quantification of intracellular Fe^2+^ content. I: Control; II: DOX/BAI NPs. *p < 0.05, **p < 0.01 vs. I group. Data are presented as mean ± SD, n = 3 for all *in vitro* experiments.

Ferroptosis is a Fe^2+^-dependent programmed cell death driven by oxidative damage. To verify whether DOX/BAI NPs could induce ferroptosis in HepG2 cells, we detected key characteristic events of ferroptosis. After cellular internalization, the acidic lysosomal microenvironment and high intracellular GSH concentration facilitate the disassembly of DOX/BAI NPs, resulting in rapid release of BAI and DOX. Released drugs further trigger intracellular ROS generation and Fe^2+^ accumulation. Excess Fe^2+^ participates in the Fenton reaction to produce hydroxyl radicals, which attack polyunsaturated fatty acids on cell membranes and promote massive lipid ROS production, accompanied by elevated MDA levels. DCFH-DA was applied to detect intracellular ROS levels, and the Calcein-AM quenching assay was used to reflect intracellular Fe^2+^ abundance. As shown in Figure 4, compared with the control group, the DOX/BAI NPs group exhibited markedly increased ROS, MDA and Fe^2+^ levels (p < 0.05 or p < 0.01). Ferroptosis arises from disrupted balance between lipid ROS generation and clearance: massive ROS accumulation together with impaired antioxidant defense finally triggers oxidative cell death, in which iron overload acts as a core driving factor. Our results demonstrated that HepG2 cells treated with DOX/BAI NPs possessed key prerequisites for ferroptosis. Therefore, we further examined other biomarkers associated with ferroptosis.

**FIGURE 5:**
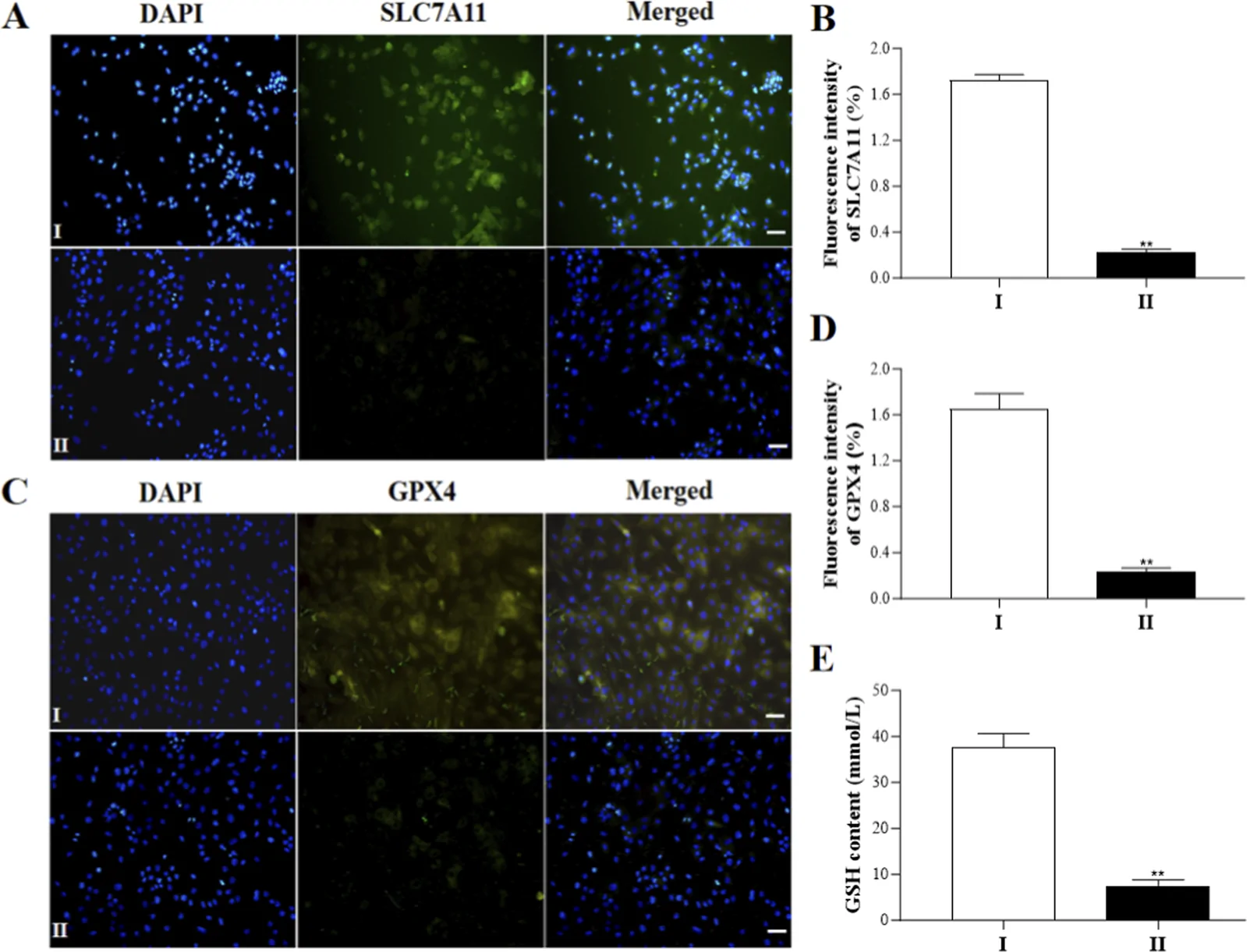
Effects of DOX/BAI NPs on SLC7A11, GPX4 protein expression and intracellular GSH content in HepG2 cells. **(A)** Representative immunofluorescence images for SLC7A11 (green) and DAPI nuclear staining. Scale bar = 20 μm. **(B)** Quantitative fluorescence intensity of SLC7A11. **(C)** Representative immunofluorescence images for GPX4 and DAPI nuclear staining. Scale bar = 20 μm. **(D)** Quantitative fluorescence intensity of GPX4. **(E)** Measurement of intracellular GSH content. I: Control; II: DOX/BAI NPs. **p < 0.01 vs. I group. Data are presented as mean ± SD, n = 3 for all *in vitro* experiments.

Ferroptosis is characterized by intracellular GSH depletion, decreased glutathione peroxidase 4 (GPX4) protein, and downregulation of solute carrier family 7 member 11 (SLC7A11). To verify these signatures, intracellular GSH content was quantified by commercial assay kits, and the protein levels of anti-ferroptosis proteins SLC7A11 and GPX4 were evaluated using immunofluorescence staining (Figure 5). Compared with the control group, the DOX/BAI NPs group exhibited significantly reduced GSH content as well as lower SLC7A11 and GPX4 protein expression. These results demonstrate that DOX/BAI NPs trigger GSH depletion, downregulate SLC7A11 and GPX4 protein levels, and impair the cellular anti-ferroptosis defense system. Collectively, these data further confirm that DOX/BAI NPs effectively induce ferroptosis in HepG2 cells.

**FIGURE 6:**
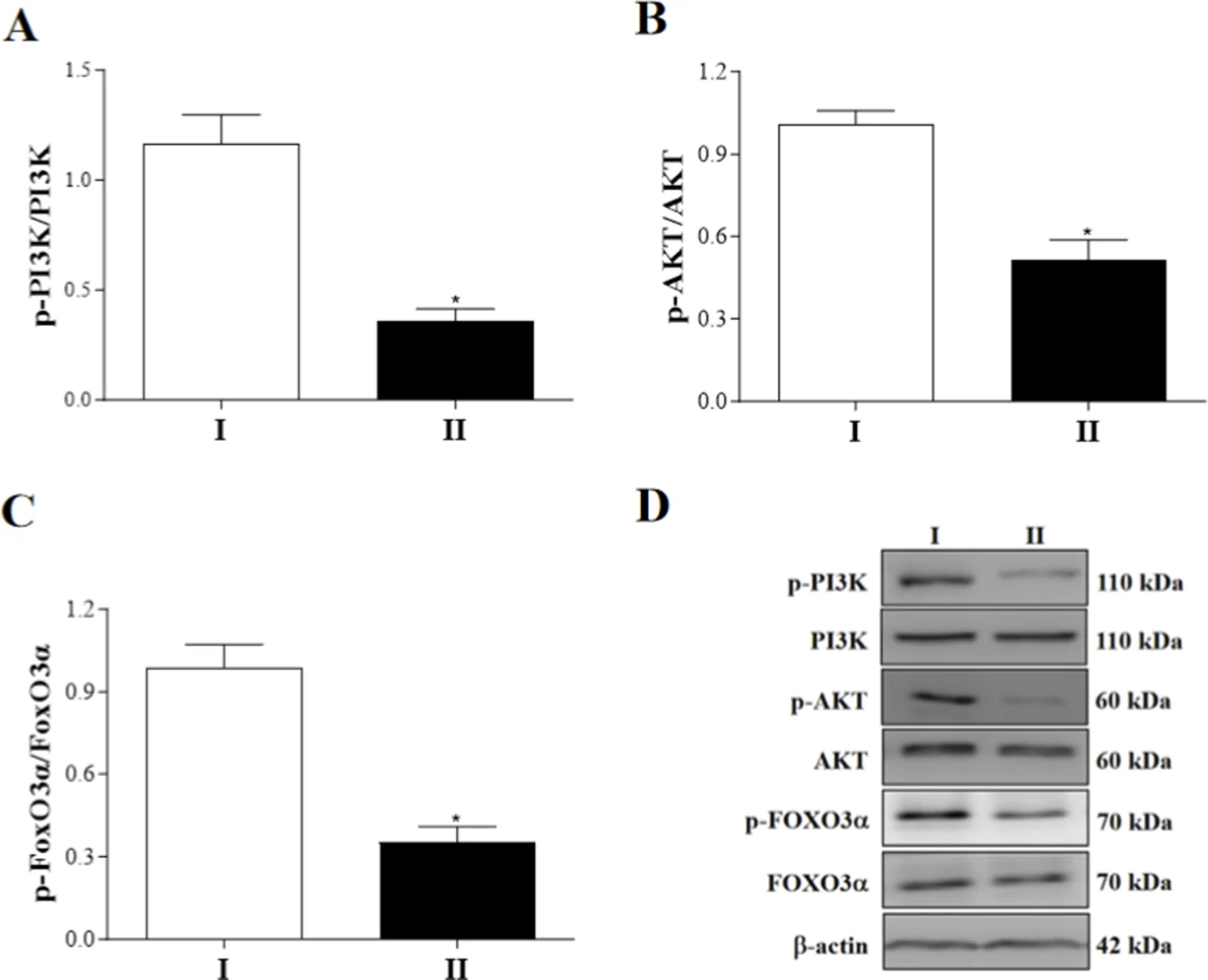
DOX/BAI NPs regulate the PI3K/AKT/FoxO3α signaling pathway in HepG2 cells. **(A)** Quantitative analysis of p-PI3K/PI3K levels. **(B)** Quantitative analysis of p-AKT/AKT levels. **(C)** Quantitative analysis of p-FoxO3α/FoxO3α levels. **(D)** Representative images depicting the expression of p-PI3K, PI3K, p-AKT, AKT, p-FoxO3α, FoxO3α, and β-actin proteins. I: Control; II: DOX/BAI NPs. *p < 0.05 vs. I group. Data are presented as mean ± SD, n = 3 for all *in vitro* experiments.

To explore the potential signaling pathway involved in DOX/BAI NPs action, we examined the phosphatidylinositol 3-kinase (PI3K)/protein kinase B (AKT)/Forkhead box O3α (FoxO3α) axis, a key survival pathway often dysregulated in cancer. Western blot analysis was employed to assess the expression levels of proteins associated with the PI3K/AKT/FoxO3α signaling pathway in HepG2 cells across different experimental groups. As illustrated in Figure 6, compared to the control group, there was a significant reduction in the protein expressions of p-PI3K, p-AKT, and p-FoxO3α in the DOX/BAI NPs group. Additionally, the ratios of p-PI3K/PI3K, p-AKT/AKT, and p-FoxO3α/FoxO3α were also markedly decreased. These findings indicate that DOX/BAI NPs effectively inhibit the activation of the PI3K/AKT/FoxO3α signaling pathway.

### 3.5 Ferroptosis induced by DOX/BAI NPs is regulated via the PI3K/AKT/FOXO3α pathway

**FIGURE 7:**
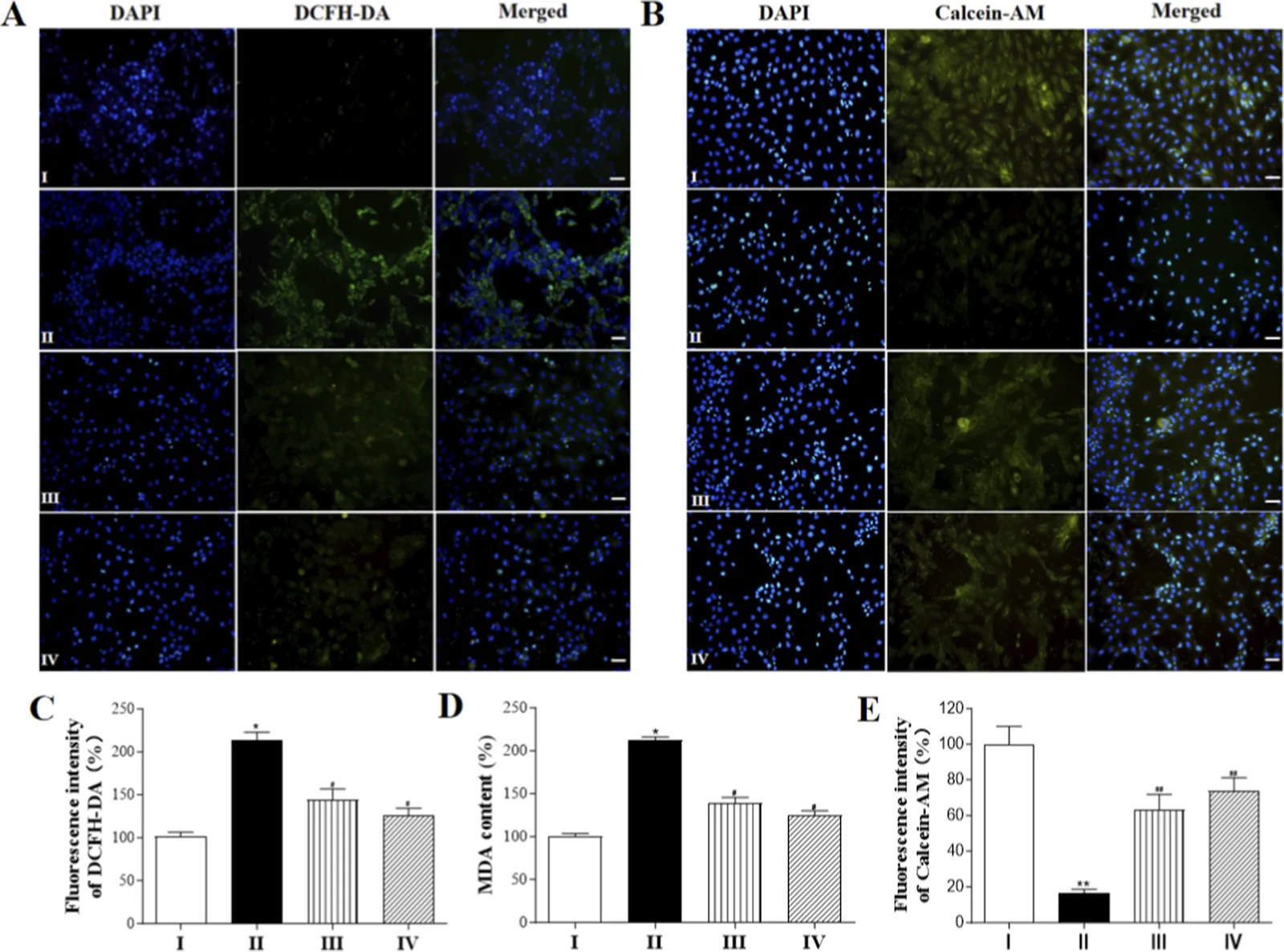
ROS, Fe^2+^ and MDA changes in DOX/BAI NPs-treated HepG2 cells with pharmacological intervention of PI3K pathway and ferroptosis. **(A)** Representative fluorescence images of DAPI and DCFH-DA staining for ROS detection. Scale bar = 20 μm. **(B)** Representative fluorescence images of DAPI and Calcein-AM staining for Fe^2+^ detection (Calcein-AM quenching assay). Scale bar = 20 μm. **(C)** Quantitative fluorescence intensity of intracellular DCFH-DA. **(D)** Intracellular MDA content. **(E)** Quantitative fluorescence intensity of Calcein-AM. I: Control; II: DOX/BAI NPs; III: SC79+DOX/BAI NPs; IV: Ferrostatin-1+DOX/BAI NPs. *p < 0.05, **p < 0.01 vs. I group; #p < 0.05, ##p < 0.01 vs. II group. Data are presented as mean ± SD, n = 3.

To verify the functional linkage between PI3K/AKT/FOXO3α signaling and nanoparticle-triggered ferroptosis, the PI3K activator SC79 was used to rescue PI3K pathway activity, and ferrostatin-1 (a classic ferroptosis inhibitor) was applied as positive control. HepG2 cells were pretreated with SC79 or ferrostatin-1 before DOX/BAI NPs treatment. As shown in Figure 7, compared with the control group (I), DOX/BAI NPs (Group II) markedly increased intracellular ROS, Fe^2+^ and MDA levels (p < 0.05 or p < 0.01). In the SC79+DOX/BAI NPs group (III), ROS, Fe^2+^ and MDA levels were significantly decreased relative to group II (p < 0.05). Similarly, ferrostatin-1 pretreatment (Group IV) also alleviated ROS accumulation, Fe^2+^ overload and lipid peroxidation. These results suggest that activation of PI3K/AKT signaling by SC79 could partially reverse DOX/BAI NPs-induced ferroptosis phenotypes, further confirming that the PI3K/AKT/FOXO3α axis participates in regulating ferroptosis triggered by DOX/BAI NPs in HepG2 cells.

**FIGURE 8:**
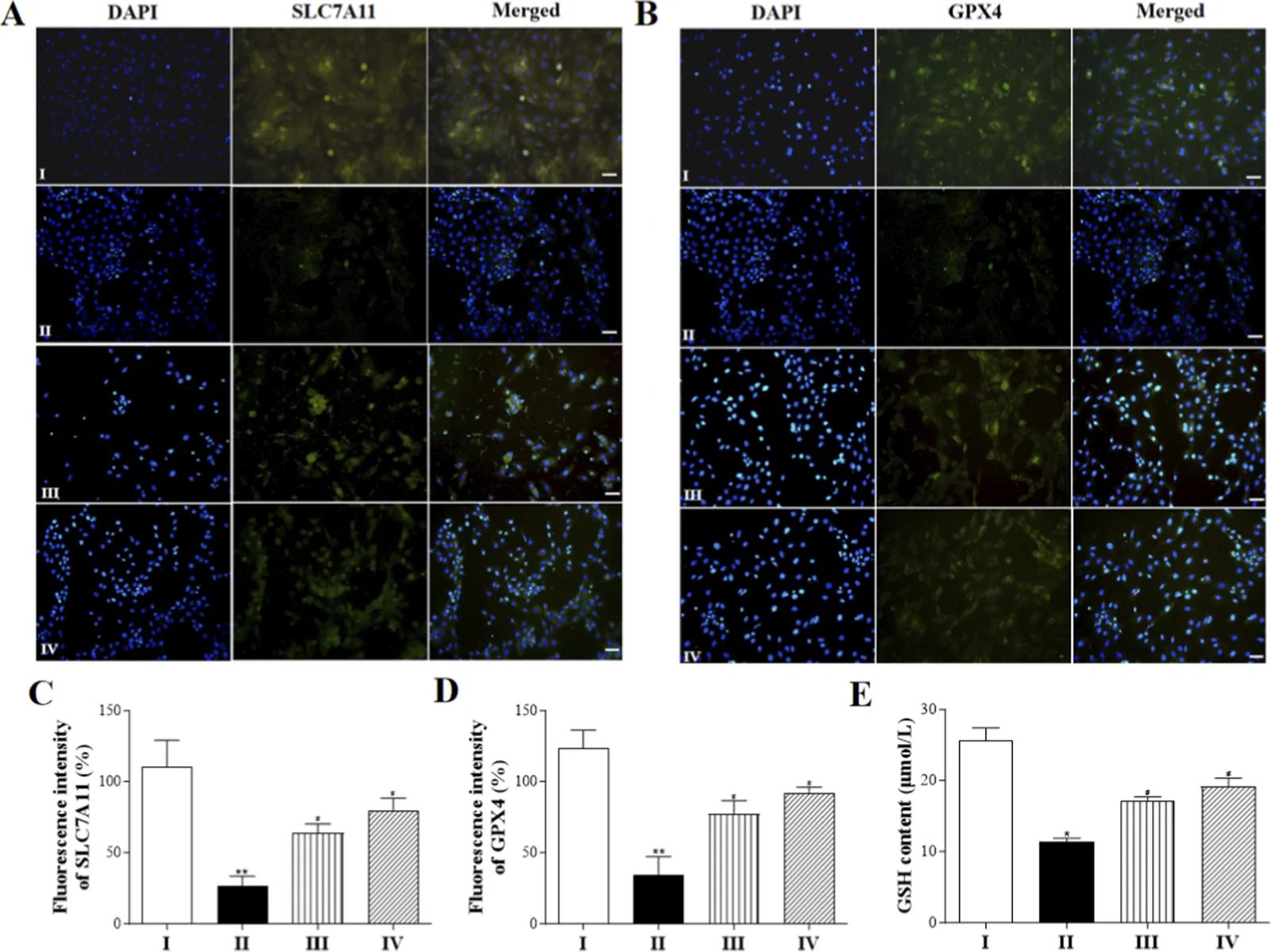
Effects of DOX/BAI NPs on SLC7A11, GPX4 protein expression and intracellular GSH content in HepG2 cells under PI3K pathway intervention. **(A)** Representative immunofluorescence images for SLC7A11 and DAPI staining. Scale bar = 20 μm. **(B)** Representative immunofluorescence images for GPX4 and DAPI staining. Scale bar = 20 μm. **(C)** Quantitative fluorescence intensity of SLC7A11. **(D)** Quantitative fluorescence intensity of GPX4. **(E)** Measurement of intracellular GSH content. I: Control; II: DOX/BAI NPs; III: SC79+DOX/BAI NPs; IV: Ferrostatin-1+DOX/BAI NPs. *p < 0.05, **p < 0.01 vs. I group; #p < 0.05 vs. II group. Data are presented as mean ± SD, n = 3.

We further detected intracellular GSH content as well as protein levels of anti-ferroptosis markers SLC7A11 and GPX4. As shown in Figure 8, relative to the control group, DOX/BAI NPs treatment markedly reduced GSH content and downregulated SLC7A11 and GPX4 protein expression (p < 0.05 or p < 0.01). After pretreatment with the PI3K activator SC79, the decreased GSH, SLC7A11 and GPX4 induced by DOX/BAI NPs were significantly restored. Similarly, ferroptosis inhibitor ferrostatin-1 also reversed these changes. Taken together, DOX/BAI NPs trigger GSH depletion and downregulation of SLC7A11 and GPX4 to induce ferroptosis, which can be rescued by PI3K activation. These results further confirm that the anti-tumor effect of DOX/BAI NPs is closely linked to the PI3K/AKT/FOXO3α signaling pathway.

### 3.6 Circulatory clearance

**FIGURE 9:**
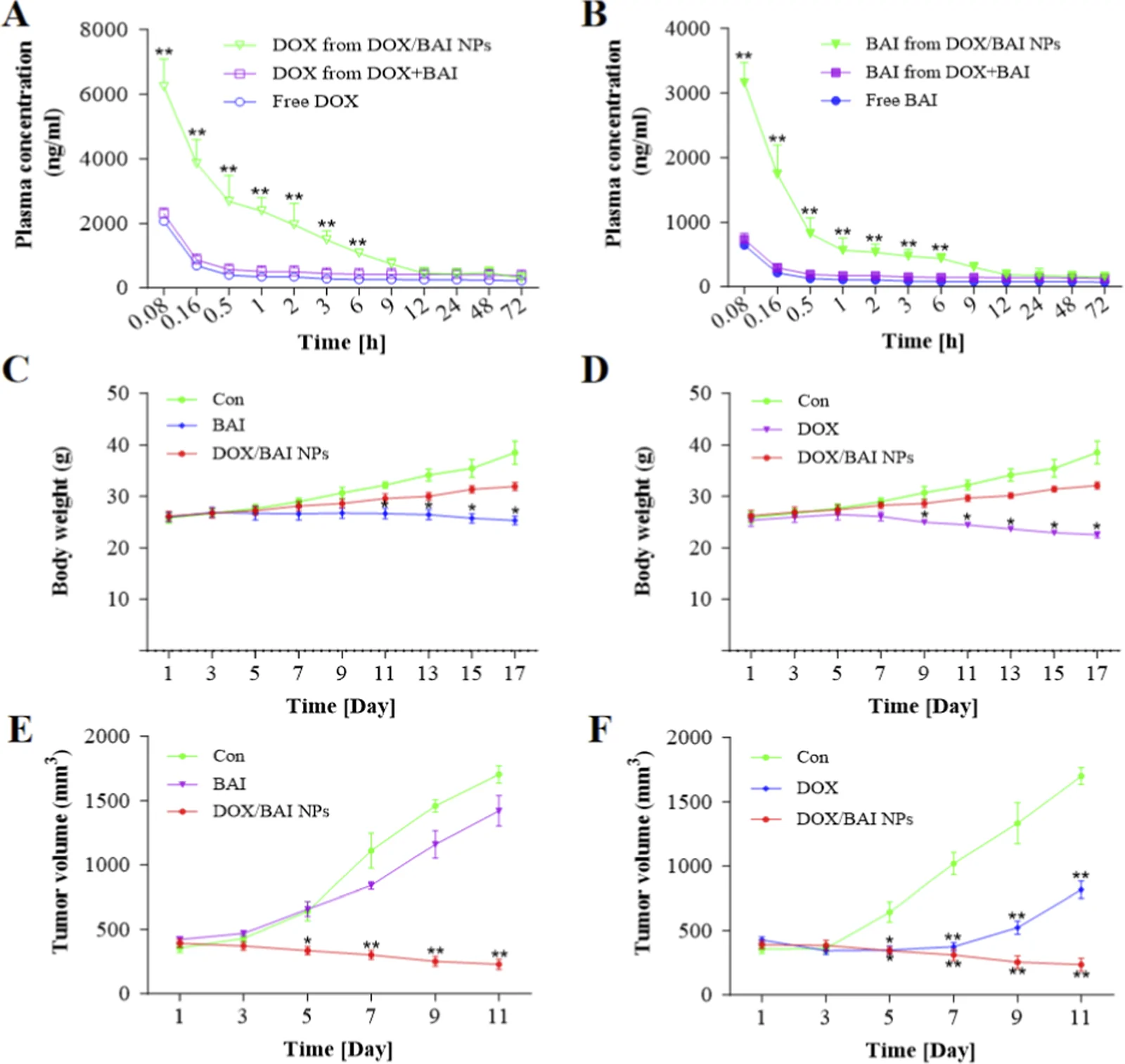
Pharmacokinetics and *in vivo* anti-tumor performance of DOX/BAI NPs. **(A)** Plasma concentration-time profiles of DOX. **(B)** Plasma concentration-time profiles of BAI after different administrations. *In vivo* safety and anti-tumor evaluation. **(C,D)** Body-weight curves of tumor-bearing mice under different treatments. **(E,F)** Tumor-volume growth curves. Statistical analysis was performed using one-way ANOVA with Tukey’s *post hoc* test at day 11. *p < 0.05, **p < 0.01. Data are presented as mean ± SD. For animal experiments, n = 5 biological replicates per group for tumor and body-weight assays; different sample size was used for plasma pharmacokinetics **(A,B)**.

Plasma concentrations of DOX and BAI were measured at predetermined time-points after intravenous injection of free DOX, free BAI, DOX + BAI mixture and DOX/BAI NPs. As shown in Figure 9A, free DOX (open circle) was rapidly cleared from circulation, with plasma concentration dropping to a low level within 0.5 h. The DOX + BAI mixture (open square) showed similar plasma DOX profiles compared with free DOX. By contrast, DOX encapsulated in DOX/BAI NPs maintained significantly higher plasma concentration within 6 h post-injection (p < 0.01). Consistent results were observed for BAI in Figure 9B. Free BAI and DOX + BAI mixture displayed fast plasma elimination, while DOX/BAI NPs remarkably prolonged BAI circulation *in vivo* within 6 h (p < 0.01).

### 3.7 *In vivo* tumor suppression assay

Body weight of tumor-bearing mice was monitored to evaluate systemic biosafety. As presented in Figures 9C,D, mice in the control group showed steady body-weight gain. The free BAI group exhibited mild body-weight loss, whereas free DOX caused more obvious weight reduction, indicating systemic toxicity of free DOX. Mice receiving DOX/BAI NPs maintained continuous body-weight increase, although the growth rate was slightly lower than control. These data demonstrate that DOX/BAI NPs have good *in vivo* biocompatibility and alleviate the systemic toxicity induced by free drugs. After cellular uptake by tumor cells, DOX/BAI NPs are disassembled in acidic lysosomes, and intracellular high-level GSH triggers disulfide-bond cleavage, accelerating co-release of DOX and BAI to exert synergistic anti-proliferative effects, thus reducing off-target toxicity.

As shown in Figures 9E,F, tumor volume continuously increased in the free BAI group but grew slower than the control group. Free DOX achieved moderate tumor suppression. Importantly, DOX/BAI NPs produced the most potent anti-tumor effect with markedly reduced tumor volume compared with both free BAI and free DOX groups. On day 11, the tumor-inhibition rate (TIR) was 29.36% for free BAI, 52.03% for free DOX, and 88.60% for DOX/BAI NPs (calculated versus control group). DOX/BAI NPs exhibited superior anti-tumor activity relative to free BAI and free DOX.

### 3.8 Histopathological safety evaluation of liver and myocardial tissues

**FIGURE 10:**
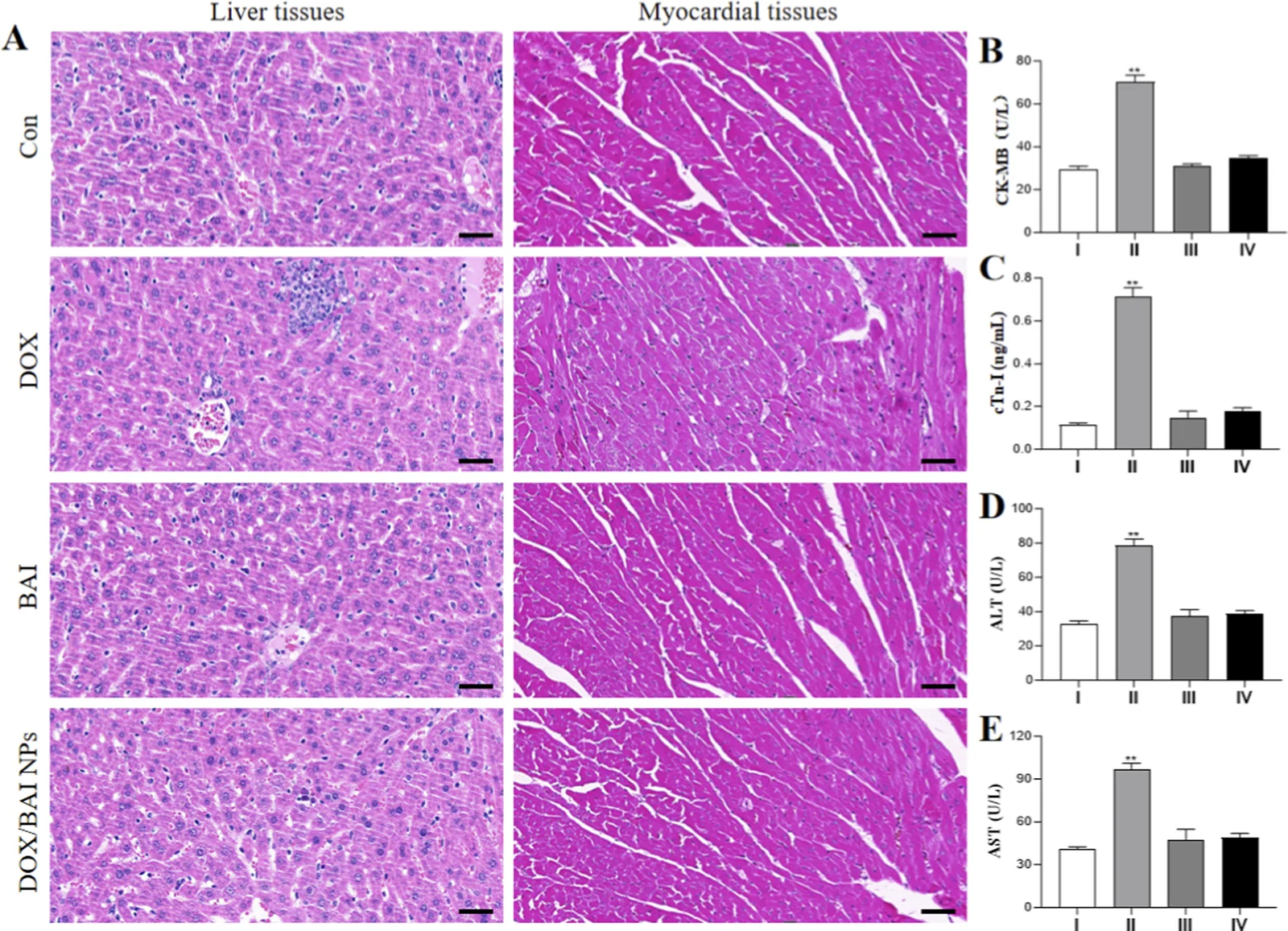
Histological features of liver and myocardial tissues and serum biochemical markers following different treatments. **(A)** Representative HE staining images of liver (left panel) and myocardial tissues (right panel). Scale bar = 50 μm. **(B–E)** Serum levels of CK-MB, cTn-I, ALT and AST. I: Control; II: free DOX; III: free BAI; IV: DOX/BAI NPs. **p < 0.01 vs. group I. Data are presented as mean ± SD, n = 5 for all *in vivo* experiments.

To further verify that DOX/BAI NPs alleviate DOX-induced systemic toxicity, HE staining was performed to examine histopathological features of liver and myocardial tissues. As shown in Figure 10, liver tissues from group I (Control) showed intact hepatic lobule architecture, neatly arranged hepatic cords and clear hepatic sinusoids. Hepatocytes exhibited normal morphology with homogeneous cytoplasm, without obvious edema, degeneration, necrosis or inflammatory cell infiltration. In group II (free DOX), mild-to-moderate hydropic degeneration of hepatocytes and sporadic inflammatory cell infiltration were observed, suggesting free-DOX-mediated hepatic injury. Group III (free BAI) retained well-preserved liver microstructure with negligible hepatocyte damage. Importantly, liver sections of group IV (DOX/BAI NPs) maintained intact hepatic lobule structure and normal hepatocyte morphology with minimal pathological lesions, indicating that the nanocarrier effectively attenuated DOX-triggered hepatotoxicity.

Consistent with liver findings, myocardial tissues in group I displayed orderly myocardial fibers, uniform cytoplasm and regular nuclei; no interstitial edema, hyperemia or inflammatory infiltration was observed. By contrast, group II showed prominent myocardial damage, including disorganized myocardial fibers, cytoplasmic vacuolar degeneration, occasional pyknotic nuclei and mild interstitial edema. For group III, myocardial structure was largely preserved, though sporadic erythrocyte exudation could be seen in the interstitium. In group IV, myocardial fibers returned to ordered arrangement, and no evident vacuolar degeneration, nuclear pyknosis or interstitial edema was detected.

Serum biochemical analyses (Figures 10B–E) demonstrated that myocardial injury markers CK-MB and cTn-I, as well as hepatic injury indicators ALT and AST, were significantly elevated in group II relative to group I (**p < 0.01). Groups III and IV exhibited markedly lower levels of these hepatic-myocardial damage biomarkers. Taken together, histopathological observations combined with serum biochemistry confirm that DOX/BAI co-loaded nanoparticles can effectively reduce DOX-elicited hepatic and myocardial adverse effects *in vivo*.

## 4 Discussion

Traditional Chinese medicine has become a promising adjuvant and alternative strategy for advanced liver cancer treatment, with abundant studies confirming that herbal active ingredients can suppress liver cancer metastasis, recurrence and drug resistance by regulating epithelial-mesenchymal transition and its upstream signaling pathways (Li et al., 2021). As a classic natural Chinese medicinal herb, Scutellaria baicalensis exerts multiple anti-tumor effects, and its core active ingredient BAI possesses excellent tumor-inhibitory activity with low cytotoxicity toward normal tissues (Duan et al., 2019; Xu et al., 2021; Shen et al., 2023). Existing studies have verified that BAI inhibits liver cancer cell proliferation in a concentration-dependent manner (Wang et al., 2014), making it an ideal natural anti-tumor candidate. However, the poor water solubility, susceptibility to photodegradation, chemical instability and low *in vivo* bioavailability of free BAI severely restrict its clinical transformation and application (Li et al., 2022). To overcome these inherent defects of single small-molecule drugs and achieve synergistic anti-HCC therapy, this study constructed a pH/GSH dual-responsive co-delivery nanoplatform (DOX/BAI NPs) based on SA, realizing synchronized delivery and tumor-targeted release of BAI and DOX. Compared with single-drug therapy and simple physical drug combination, this dual-responsive nano-delivery system exhibits unique superiority in synergistic anti-tumor efficacy, mitigation of toxic side-effects and clinical translational potential, which is systematically discussed below.

In this work, the amphiphilic polymer SA-SS-BAI was successfully synthesized via CYS cross-linking, which self-assembles into uniform spherical nanoparticles in aqueous solution and efficiently loads DOX. The SA carrier endows the nanosystem with excellent biocompatibility and pH responsiveness owing to abundant hydroxyl and carboxylate groups in its molecular structure (Sun et al., 2024), while disulfide bonds from CYS serve as TME-responsive switches that can be specifically cleaved by high-concentration GSH in tumor tissues, triggering nanoparticle disassembly and accelerated drug release (Zhang J et al., 2024). Material characterization confirmed that DOX/BAI NPs possess uniform particle size (∼148 nm), satisfactory drug-loading capacity and favorable colloidal stability. Consistent with our design expectation, nanoparticles remain stable under normal physiological conditions to prevent premature drug leakage, yet rapidly disassemble and release payloads in acidic, high-GSH TME, achieving tumor-specific drug delivery. *In vitro* cellular assays further demonstrated that DOX/BAI NPs achieve markedly higher cellular internalization in HepG2 cells than free drugs or physical mixtures; cellular uptake proceeds via energy-dependent caveolae-mediated endocytosis. This endocytic route helps evade severe lysosomal degradation, guarantees efficient intracellular drug liberation and strengthens cytotoxicity against HCC cells.

Notably, the core advantage of this SA-based dual-responsive co-delivery system lies in spatiotemporally synchronized co-delivery and the molecular-level synergistic anti-tumor mechanism of BAI and DOX. Free BAI combined with DOX only generates limited *in vitro* anti-proliferative synergy, and simple physical mixtures fail to exert effective therapeutic effects *in vivo* because the two agents display divergent pharmacokinetic profiles and tissue distribution. In contrast, the nanocarrier integrates BAI and DOX within one single nano-construct, enabling simultaneous intracellular drug delivery within the same tumor cell, prolonged systemic circulation and TME-triggered on-demand drug release. More importantly, DOX/BAI NPs substantially alleviate the severe myocardial and hepatic toxicity that represents a major clinical bottleneck of free-DOX chemotherapy, an outcome that cannot be accomplished by simply mixing free BAI and DOX. This highlights the irreplaceable value of the dual-responsive co-delivery nanoplatform for improving the therapeutic index of chemotherapeutic agents.

Mechanistically, ferroptosis induction constitutes the major anti-tumor mode-of-action for DOX/BAI NPs. Ferroptosis is an iron-dependent programmed cell death driven by lipid peroxidation, tightly governed by intracellular GSH content, ROS abundance, Fe^2+^ overload, as well as key functional proteins including SLC7A11 and GPX4 (Dixon et al., 2012; Song et al., 2018; Bersuker et al., 2019). As the critical cysteine importer, SLC7A11 sustains GSH homeostasis; repression of the SLC7A11/GPX4 axis triggers GSH exhaustion and subsequent ferroptosis (Gao et al., 2021). Previous reports have illustrated that both DOX and BAI can independently modulate ferroptosis: DOX boosts ROS burst and Fe^2+^ accumulation through iron-dependent Fenton reactions, whereas BAI suppresses system xc^−^ and GPX4 antioxidant function (Wang et al., 2021; Wen et al., 2023; Zhang Z et al., 2025). In our present study, DOX/BAI NPs significantly downregulated SLC7A11 and GPX4 protein expression, depleted intracellular GSH, and elevated Fe^2+^ and ROS levels in HepG2 cells. This co-delivery nanosystem combines the ferroptosis-promoting effects of BAI and DOX, producing dual disruption of tumor antioxidant defense and cellular iron homeostasis, thereby eliciting robust ferroptotic cell death that cannot be readily achieved by single-drug treatment.

Further mechanistic exploration identified the PI3K/AKT/FOXO3α signaling axis as an essential upstream regulator of DOX/BAI NPs-driven ferroptosis. The PI3K/AKT cascade is a core pro-survival pathway frequently dysregulated in HCC, and its aberrant activation correlates strongly with ferroptosis resistance in tumors (Revathidevi and Munirajan, 2019; Lv et al., 2024; Luo et al., 2024). Multiple studies have documented that PI3K/AKT inactivation modulates FOXO3α transcriptional activity, further altering downstream iron-metabolism and redox-related gene expression and shaping tumor ferroptosis susceptibility (Liang et al., 2019; Wang S et al., 2023; Wang et al., 2024). Consistent with earlier literature, our western blot results revealed that DOX/BAI NPs remarkably decrease phosphorylation of PI3K, AKT and FOXO3α in HepG2 cells, indicating effective suppression of the PI3K/AKT/FOXO3α pro-survival pathway. Critically, pharmacological rescue experiments using the PI3K activator SC79 partially restored ferroptosis-associated phenotypes provoked by DOX/BAI NPs, including recovery of SLC7A11/GPX4 expression, replenished GSH, and reduced Fe^2+^ and ROS levels. These causal pharmacological evidences demonstrate that inhibition of the PI3K/AKT/FOXO3α axis is not merely a secondary by-product, but a key upstream event contributing to nanoparticle-initiated ferroptosis. Suppression of this oncogenic pathway remodels FOXO3α-dependent transcription, disturbs intracellular redox and iron homeostasis, and synergistically amplifies BAI- and DOX-mediated ferroptosis, forming a multi-layered synergistic anti-tumor mechanism.

From the perspective of delivery-system innovation and translational prospect, this SA-based dual-responsive nanoplatform possesses prominent merits compared with conventional nano-formulations. First, the natural marine polysaccharide SA serves as the carrier, featuring good biocompatibility, biodegradability and low immunogenicity, circumventing biosafety risks associated with synthetic polymers and supporting future translational development. Second, BAI functions simultaneously as the hydrophobic moiety of the amphiphilic polymer and therapeutic payload, removing extra hydrophobic modification procedures, simplifying material synthesis and improving drug-loading performance. Third, pH/GSH dual-responsiveness enables tumor-selective drug activation: nanoparticles remain stable in normal tissues with neutral pH and low GSH to prevent off-target payload leakage, while disassembling specifically within acidic high-GSH TME to execute anti-tumor effects, establishing a favorable therapeutic window.

*In vivo* animal experiments further validated the superior anti-tumor efficacy and biosafety of DOX/BAI NPs. Benefiting from the nano-size effect, DOX/BAI NPs prolong systemic circulation lifetime of both drugs and enhance tumor accumulation via the EPR effect. TME-triggered payload release maintains high intra-tumoral drug concentration, and PI3K/AKT/FOXO3α-mediated ferroptosis synergism endows the nanosystem with potent *in vivo* anti-tumor activity. For biosafety evaluation, free DOX causes severe myocardial and hepatic injury due to non-specific distribution and oxidative damage, reflected by elevated serum myocardial/hepatic injury biomarkers (CK-MB, cTn-I, ALT, AST) and evident pathological lesions in liver and myocardium. In contrast, DOX/BAI NPs markedly mitigate DOX-related systemic toxicity and preserve normal body-weight gain in mice. On one hand, the dual-responsive nanocarrier avoids premature DOX leakage during blood circulation and lowers drug exposure of healthy organs; on the other hand, synchronously delivered BAI confers antioxidant and cytoprotective effects on normal tissues. Notably, the simple physical combination of free DOX and BAI cannot achieve comparable protective outcomes, confirming that nanoparticle-mediated synchronous co-delivery is indispensable for balancing anti-tumor potency and normal-tissue protection.

Limitations of the present study should also be acknowledged. All *in vivo* antitumor evaluations were performed using subcutaneous H22 hepatoma xenograft models, which cannot fully recapitulate the complex tumor microenvironment of orthotopic HCC. In addition, pathway validation in this work relied primarily on pharmacological intervention with SC79; further genetic manipulation of FoxO3α is required to consolidate the causal relationship between this axis and nanoparticle-triggered ferroptosis in HCC cells. These points will be addressed in our follow-up investigations.

## 5 Conclusion

**FIGURE 11:**
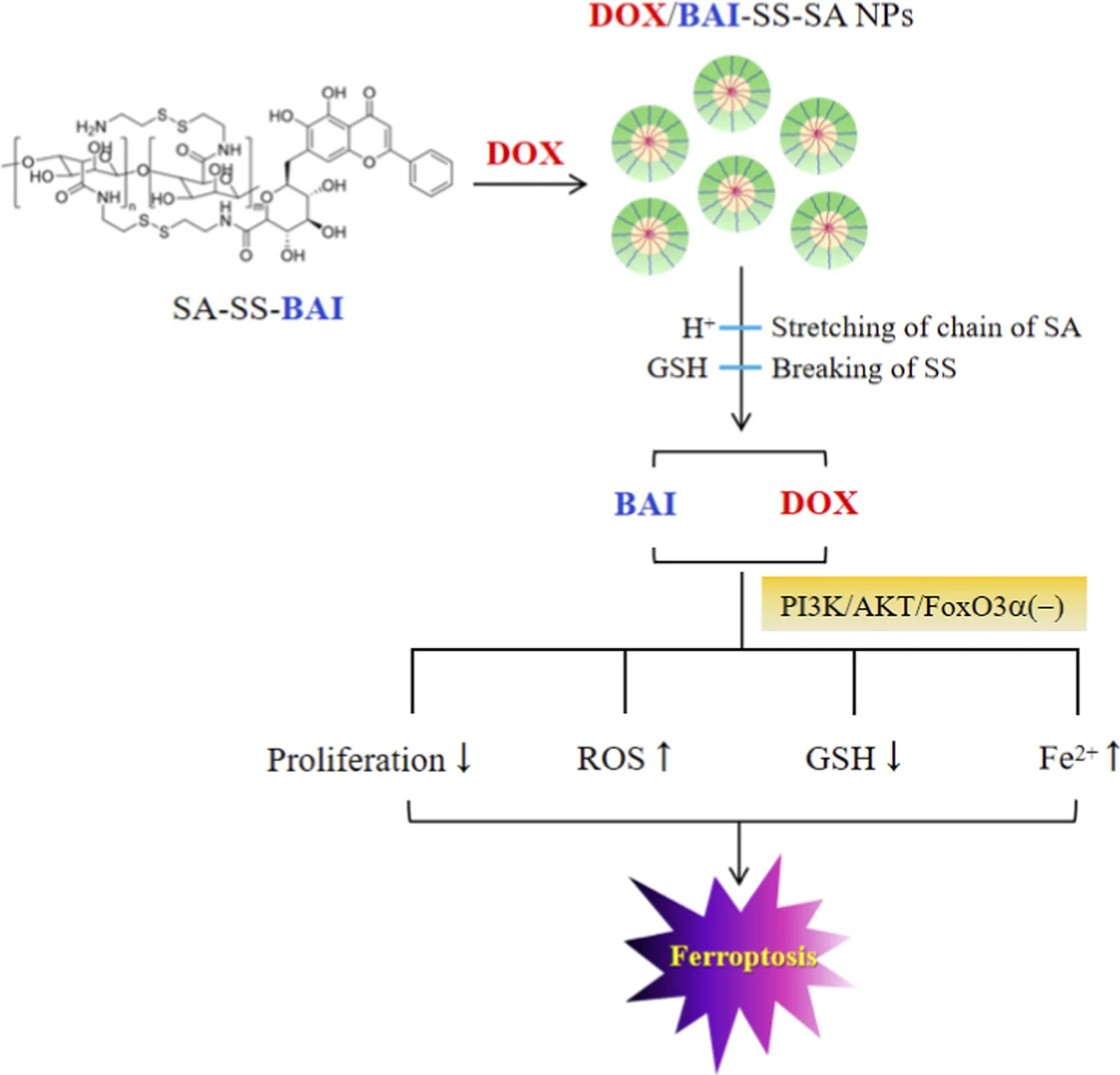
Schematic illustration depicting the synthesis, tumor-microenvironment-triggered disassembly, and anti-HCC ferroptotic mechanism of DOX/BAI-co-loaded SA-SS-BAI dual-responsive nanoparticles. The amphiphilic SA-SS-BAI polymer self-assembles and encapsulates DOX to form spherical nanoparticles. Following tumor enrichment via the EPR effect in HCC tissue, acidic H^+^ stretches sodium alginate chains, while high intracellular GSH cleaves disulfide (-SS-) bonds, triggering rapid release of BAI and DOX. The released BAI together with DOX synergistically suppresses the PI3K/AKT/FOXO3α signaling axis. This cascade inhibits HCC cell proliferation, increases intracellular ROS and Fe^2+^ levels, and depletes cellular GSH. Collectively, these metabolic disturbances initiate robust ferroptosis and suppress hepatocellular carcinoma progression.

In summary, this study systematically illustrates the unique advantages and research value of the DOX/BAI co-loaded dual-responsive nano-delivery system. Distinct from monotherapy and physical drug combinations, this nanoplatform achieves precise spatiotemporal co-delivery of dual agents, induces complementary ferroptosis through BAI-mediated SLC7A11/GPX4 suppression and DOX-driven Fenton reaction, and further augments synergistic anti-tumor efficacy via inhibiting the PI3K/AKT/FOXO3α pathway. Meanwhile, natural SA matrix guarantees good biosafety and translational potential, and tumor-specific responsive release resolves the systemic toxicity drawback of DOX chemotherapy. This work provides a novel and safe synergistic therapeutic strategy for HCC, and offers new references for designing co-delivery nanosystems combining natural herbal ingredients and chemotherapeutic drugs (Figure 11).

## Data Availability

The original contributions presented in the study are included in the article/Supplementary Material, further inquiries can be directed to the corresponding author.
