# Supplementary material for: Sodium alginate-based dual-responsive nanoparticles co-loaded with baicalin and doxorubicin induce ferroptosis in hepatocellular carcinoma associated with the PI3K/AKT/FoxO3α axis regulation
Source: Front Cell Dev Biol. 2026 Sep 3;14:1905104. doi: 10.3389/fcell.2026.1905104 (PMC13582609; doi:10.3389/fcell.2026.1905104)
Supplement: Supplementary file 1 [file Table1.docx]

Table S1

| μg/mL | I vs II | I vs III | I vs IV | II vs III | II vs IV | III vs IV |
| --- | --- | --- | --- | --- | --- | --- |
| 0.25 | ** | ** | ** | * | ns | ns |
| 0.5 | ** | ** | ** | ns | ns | ns |
| 1 | ** | ** | ** | * | * | ns |
| 5 | ** | ** | ** | * | ** | * |
| 10 | ** | ** | ** | * | ** | * |

Table S2

| μg/mL | I vs II | I vs III | I vs IV | II vs III | II vs IV | III vs IV |
| --- | --- | --- | --- | --- | --- | --- |
| 0.25 | ** | ** | ** | ns | ns | ns |
| 0.5 | ** | ** | ** | ns | ns | ns |
| 1 | ** | ** | ** | ns | ns | ns |
| 5 | ** | ** | ** | ns | * | * |
| 10 | ** | ** | ** | ns | * | * |

Table S3

| μg/mL | I vs II | I vs III | I vs IV | II vs III | II vs IV | III vs IV |
| --- | --- | --- | --- | --- | --- | --- |
| 0.25 | ** | ** | ns | ns | ** | ** |
| 0.5 | ** | ** | ns | ns | ** | ** |
| 1 | ** | ** | ns | ns | ** | ** |
| 5 | ** | ** | * | ns | ** | ** |
| 10 | ** | ** | * | ns | ** | ** |
